# Curcumin Regulates the r(CGG)^exp^ RNA Hairpin Structure and Ameliorate Defects in Fragile X-Associated Tremor Ataxia Syndrome

**DOI:** 10.3389/fnins.2020.00295

**Published:** 2020-04-07

**Authors:** Arun Kumar Verma, Eshan Khan, Subodh Kumar Mishra, Amit Mishra, Nicolas Charlet-Berguerand, Amit Kumar

**Affiliations:** ^1^Discipline of Biosciences and Biomedical Engineering, Indian Institute of Technology Indore, Indore, India; ^2^Cellular and Molecular Neurobiology Unit, Indian Institute of Technology Jodhpur, Rajasthan, India; ^3^Translational Medicine and Neurogenetics, Institut de Génétique et de Biologie Moléculaire et Cellulaire (IGBMC), INSERM U964, CNRS UMR7104, University of Strasbourg, Strasbourg, France

**Keywords:** FXTAS, therapeutics, trinucleotide, CGG repeat, RAN translation, splicing defects

## Abstract

Fragile X-associated tremor ataxia syndrome is an untreatable neurological and neuromuscular disorder caused by unstable expansion of 55–200 CGG nucleotide repeats in 5′ UTR of Fragile X intellectual disability 1 (*FMR1*) gene. The expansion of CGG repeats in the *FMR1* mRNA elicits neuronal cell toxicity through two main pathogenic mechanisms. First, mRNA with CGG expanded repeats sequester specific RNA regulatory proteins resulting in splicing alterations and formation of ribonuclear inclusions. Second, repeat-associated non-canonical translation (RANT) of the CGG expansion produces a toxic homopolymeric protein, FMRpolyG. Very few small molecules are known to modulate these pathogenic events, limiting the therapeutic possibilities for FXTAS. Here, we found that a naturally available biologically active small molecule, Curcumin, selectively binds to CGG RNA repeats. Interestingly, Curcumin improves FXTAS associated alternative splicing defects and decreases the production and accumulation of FMRpolyG protein inclusion. Furthermore, Curcumin decreases cell cytotoxicity promptly by expression of CGG RNA in FXTAS cell models. In conclusion, our data suggest that small molecules like Curcumin and its derivatives may be explored as a potential therapeutic strategy against the debilitating repeats associated neurodegenerative disorders.

## Graphical Abstract

Candidate small molecule Curcumin ameliorates expanded CGG RNA mediated disease pathogenesis.

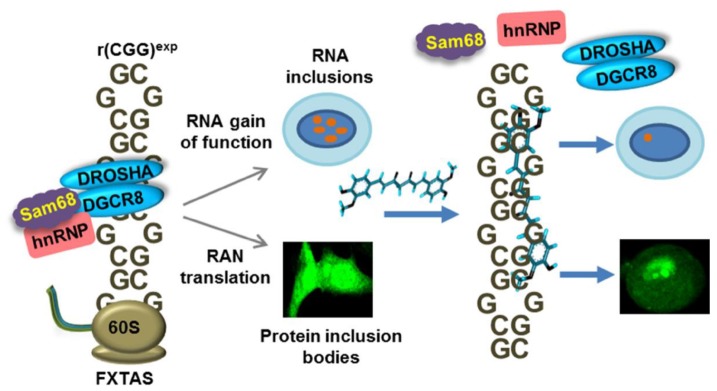

## Introduction

Many, if not all, cellular functions are controlled by changes in mRNA or non-coding RNA expression and regulations (Sharp, 2009). Thus, alterations in RNA metabolism can lead to many pathologies, including neuro-muscular disorders and cancer (Wilton and Fletcher, 2005; Calin and Croce, 2006). In that aspect, expression of RNA containing trinucleotide repeats (TNR) expansion at different gene locations such as 5′ untranslated regions, exons, introns, and 3′ untranslated regions causes various disorders, like, Fragile X-associated tremor ataxia syndrome (FXTAS), Fragile X-associated primary ovarian insufficiency (FXPOI) (Fraint et al., 2014; Groh et al., 2014), Huntington’s disease (HD) (Urbanek et al., 2017), Spinocerebellar ataxia type 10 (SCA10) (Bushara et al., 2013) Amyotrophic lateral sclerosis (ALS) (Balendra and Isaacs, 2018), Huntington’s disease-like 2 (HDL2) and Myotonic dystrophy type 1 (DM1) (López-Morató et al., 2018). These repeats expansion (CGG, CAG, GGGGCC, AUUCU, CCUG, CUG, etc.) elicit neuronal toxicity *via* overlapping pathogenic mechanisms such as toxic protein and RNA gain-of-function, and protein loss-of-function (Orr and Zoghbi, 2007; Nageshwaran and Festenstein, 2015; Freibaum and Taylor, 2017). Specifically, FXTAS is a monogenic late onset neurodegenerative disorder that affects older males and females and caused by an expansion of 55–200 CGG trinucleotide repeats in the 5′ UTR of the Fragile X mental retardation 1 (*FMR1*) gene that encodes the Fragile X mental retardation protein (FMRP) (Hagerman and Hagerman, 2015, 2016). Clinical characteristics of FXTAS includes intention tremor, parkinsonism, dysautonomia, and cerebellar and action gait ataxia (Berry-Kravis et al., 2007; Coffey et al., 2008). Of interest, CGG repeats longer than 200 trigger CpG methylation that results in epigenetic silencing of the *FMR1* gene and subsequently decrease expression of the FMR protein, leading to a different pathology, the neurodevelopmental Fragile X syndrome characterized by autism and intellectual disability.

CGG repeats in FXTAS are pathogenic through two main mechanisms, RNA gain of function and protein gain of function. First, transcribed CGG repeats RNA form stable hairpin structure that sequester specific RNA biogenesis proteins such as Src-associated in mitosis of 68 kDa (Sam68) (Sellier et al., 2010), DiGeorge syndrome critical region 8 (DGCR8) (Sellier et al., 2013), TAR DNA-binding protein (TDP-43) (He et al., 2014) and heterogeneous nuclear ribonucleoprotein (hnRNP A2/B1) (Sofola et al., 2007), which regulate pre-mRNA alternative splicing. Sequestration of these RNA binding proteins by the expanded CGG RNA repeats causes specific splicing defects and formation of ribonuclear inclusion bodies (Iwahashi et al., 2006; Sellier et al., 2010, 2013). A second pathogenic mechanism in FXTAS is the initiation of protein translation without using start codon by the expanded CGG repeats and produce toxic proteins (Todd et al., 2013; Kearse et al., 2016; Sellier et al., 2017). CGG repeats are notably translated through initiation at non-canonical ACG and GUG start codons into a polyalanine (FMRpolyA), and polyglycine (FMRpolyG), containing homopolymeric protein aggregates, which are found in positive intranuclear inclusions in FXTAS cell and mouse models as well as in FXTAS patient tissues (Todd et al., 2013; Buijsen et al., 2014; Sellier et al., 2017). These protein aggregates are responsible for neurodegeneration and disease phenotypes (Cleary et al., 2018). Importantly, genome wide studies reported that RANT is not a rare process, it occurs in many cellular processes (Pearson, 2011; Zu et al., 2011). Thus, designing a small molecule that inhibits the RAN translation event could be used to study the importance of such translations processes in the cell. Given their roles in multiple disorders, repeat containing RNAs are an attractive target for chemical probe and drug development (Warner et al., 2018; Verma et al., 2019a). However, there are very few drugs designed against RNA yet. In this study, we focused on the identification of small molecules targeting expanded CGG RNA repeats [r(CGG)^exp^] causing FXTAS. Ideally, a potential compound would prevent non-canonical translation of the CGG repeats sequence into the toxic FMRpolyG protein, as well as correct the alternative splicing defects caused by sequestration of RNA regulatory proteins by expanded CGG nucleotide (Yang et al., 2015, 2016b; Verma et al., 2019b). Here, we found that Curcumin, a polyphenol used as a traditional herbal medicine, binds to CGG RNA repeats (Nafisi et al., 2009; Kulkarni and Dhir, 2010). We validated the binding potential and selectivity of Curcumin to multiple CGG repeat RNAs using different biophysical techniques such as isothermal calorimetry titration (ITC), CD melting assay, circular dichroism (CD) spectroscopy, gel shift and PCR stop assays. Importantly, Curcumin corrects pre-mRNA alternative mis-splicing defects caused by expanded CGG repeats expression. Furthermore, Curcumin also reduces the formation of FMRpolyG protein aggregates and improves cell viability in FXTAS cell models. In conclusion, our study suggests that Curcumin could be considered as a probable small molecule for a therapeutic approach in the neurodegenerative FXTAS and FXPOI.

## Results

### Binding Affinity and Selectivity of Curcumin With Different Trinucleotide RNA Repeats

There is high demand to develop therapeutic approaches targeting the molecular mechanisms causing FXTAS pathogenesis. As small molecules that interact with r(CGG)^exp^ RNA are able to reverse the toxic consequences of CGG expansions. We searched for naturally available and biologically potent small molecules that would bind to CGG RNA repeat (Nafisi et al., 2009).

**FIGURE 1:**
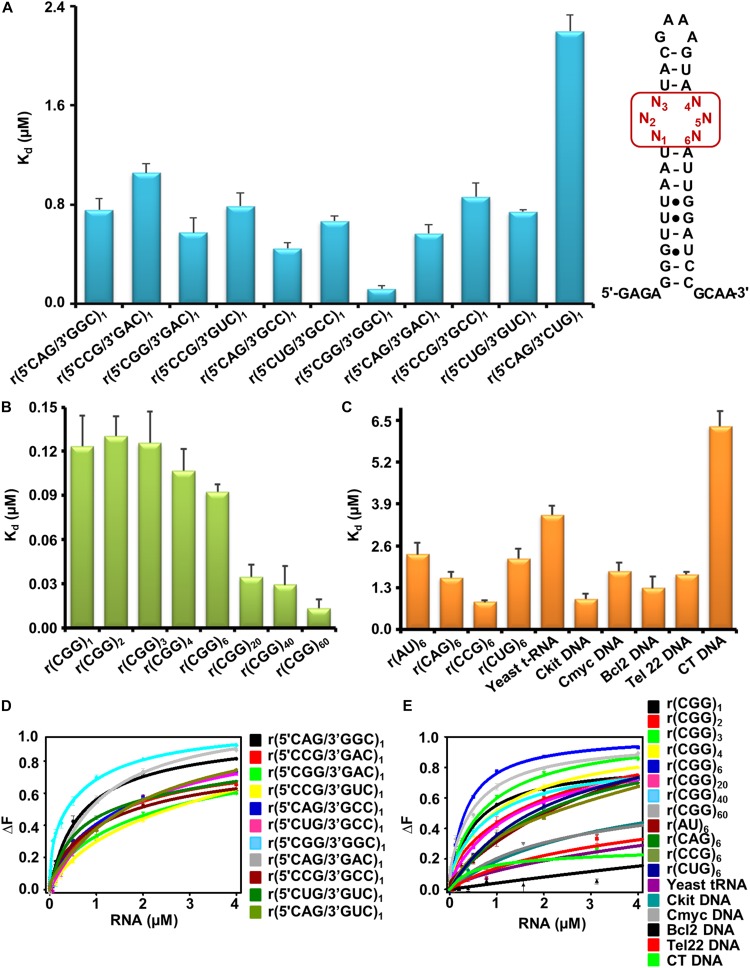
Elucidation of binding affinity (K_d_) of Curcumin with nucleic acids using fluorescence based assay. **(A)** Bar graph representing the binding constant (K_d_) values of Curcumin with different 1 × 1 nucleotide internal motif RNAs. **(B)** Bar graph showing binding affinities (K_d_) of Curcumin with different expanded CGG RNAs. **(C)** Bar chart showing K_d_ values of Curcumin with various RNA and DNA controls. **(D)** Plot illustrates curves fitting for fluorescence binding assay of Curcumin with different 1 × 1 nucleotide internal loop RNAs. **(E)** Plot illustrates curves fitting for fluorescence binding assay of Curcumin with different CGG repeat containing RNAs along with different RNA and DNA controls. The Curve was fitted using two mode binding model.

As the first step, we determined the binding selectivity and affinity of Curcumin for different trinucleotide repeats RNAs with 5′CNG/3′GNCx1 sequence where N stands for either G, C, A, and U bases (Supplementary Figure S1). Natural fluorescence of Curcumin molecule was utilized to develop a fluorescence based binding assay recorded at the emission maxima 496 nm in the presence or absence of different repeat containing RNAs. With the gradual addition of RNA to Curcumin solution, an enhancement in fluorescence intensity was detected revealing a preferential interaction of Curcumin with CGG RNA motif (Figures 1A,D, Supplementary Figure S2, and Supplementary Table S1). To confirm the binding affinity of Curcumin to CGG expanded RNA repeats, we performed the fluorescence titration assay with higher CGG repeats RNAs includes r(CGGx6) r(CGGx20), r(CGGx40), and r(CGGx60) RNA. Interestingly, Curcumin showed ∼62 and 161 folds better binding for r(CGGx40) and r(CGGx60) RNA over r(AUx1) duplex pair RNA, respectively (Figures 1B–E, Supplementary Figure S3, and Supplementary Table S2). As further controls, binding assay were also performed with r(AUx6) duplex RNA and yeast t-RNA. Curcumin exhibit ∼178 and 273 folds tighter binding with r(CGGx60) RNA over r(AUx6) and yeast t-RNA, respectively (Figures 1C–E, Supplementary Figure S3, and Supplementary Table S2). It has been described that expanded CUG, CAG, and CCG nucleotide repeats in the transcripts lead to dysregulation of the splicing process and toxic protein aggregates formation in neuronal cells (Green et al., 2016). Similar to 1 × 1 GG motif in r(CGG)^exp^, UU, CC, and AA hairpin loop structures are formed in r(CUG)^exp^, r(CCG)^exp^, and r(CAG)^exp^ RNAs. Binding studies demonstrated that Curcumin showed high selectivity with CGG RNA over other mismatched RNA. Finally, different G-quadruplexes forming DNA (*c-kit, cmyc, tel22 and bcl2*) and duplex calf thymus (CT) DNA controls were also tested (Figures 1C–E, Supplementary Figure S3, and Supplementary Table S2). Binding studies revealed that Curcumin binds a hundred folds better with CGG RNA than duplex DNA, supporting a specificity of Curcumin for CGG motif RNA in the nanomolar range.

### Isothermal Calorimetry Titration of Curcumin With CGG Repeats RNA

**FIGURE 2:**
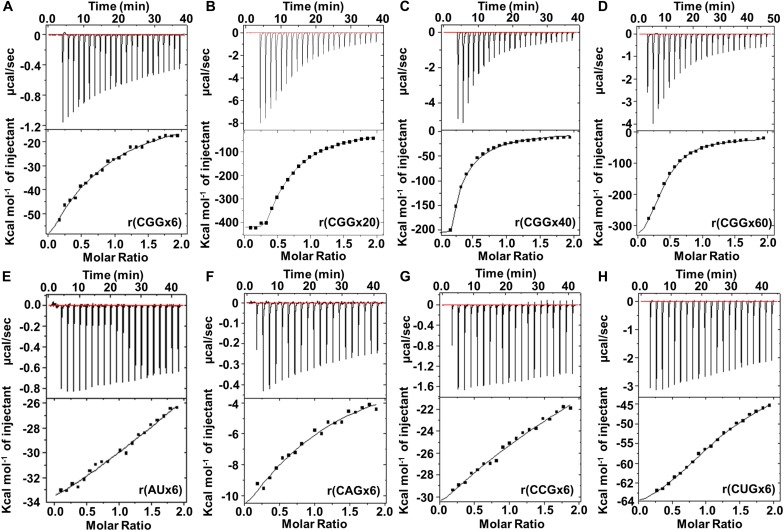
Isothermal calorimetry titration of Curcumin with different mismatched and fully paired RNAs. **(A)** r(CGGx6), **(B)** r(CGGx20), **(C)** r(CGGx40), **(D)** r(CGGx60) and **(E)** r(AUx6), **(F)** r(CAGx6), **(G)** r(CCGx6), and **(H)** r(CUGx6) represents the thermogram of respective RNA with Curcumin. The exothermic peaks represent the favorable contribution of energy during interactions. Two-mode ligand binding saturation model was used to fit the plots.

The binding affinity of Curcumin to total yeast t-RNA is in the micromolar range (Nafisi et al., 2009) while we observed a much higher affinity toward CGG repeats RNA (Figure 1). To confirm the selectivity and affinity of this interaction, we performed Isothermal calorimetry (ITC) experiments that calculate thermodynamics of non-covalent interaction between two molecules such as nucleic acid-ligand (Falconer and Collins, 2011). The thermodynamic parameters include like stoichiometry (N), change in enthalpy (ΔH), change in entropy (ΔS), dissociation constant (K_d_) and association constant (K_a_) were derived using Origin 7.0 software (Supplementary Table S3). ITC results (Figure 2) showed negative change in enthalpy for Curcumin-CGG RNA interaction, which indicate favorable interactions (Freyer and Lewis, 2008). Furthermore, exothermic peaks throughout titration imply an intercalation mode of binding of Curcumin due to π-π stacking interaction with RNA bases (Figures 2A–D; Giri and Kumar, 2008; Das et al., 2011).

The value of association constant (*K*_a_) of the highest affinity binding sites for r(CGGx6), r(CGGx20), r(CGGx40) and r(CGGx60) RNAs are 1.7 × 10^6^ M^–1^, 3.8 × 10^7^ M^–1^, 8.5 × 10^7^ M^–1^, and 8.6 × 10^7^ M^–1^, respectively (Supplementary Table S3). These data confirm that increase in repeat length of CGG RNA enhances the binding affinity of Curcumin. As a control, Curcumin showed lower association constant with other mismatched RNAs [r(CAGx6), r(CCGx6), r(CUGx6)] and AU paired duplex RNA (Figures 2E–H). Thus, Curcumin binds 24, 535, 1197, and 1211 fold more tightly to r(CGGx6), r(CGGx20), r(CGGx40), and r(CGGx60) RNAs, respectively over AU duplex RNA. Overall, ITC experiments complement fluorescence titration binding assays and evidently confirmed the interaction of Curcumin with CGG repeats RNAs.

### Curcumin Induced Conformational Changes in CGG Repeats RNA

**FIGURE 3:**
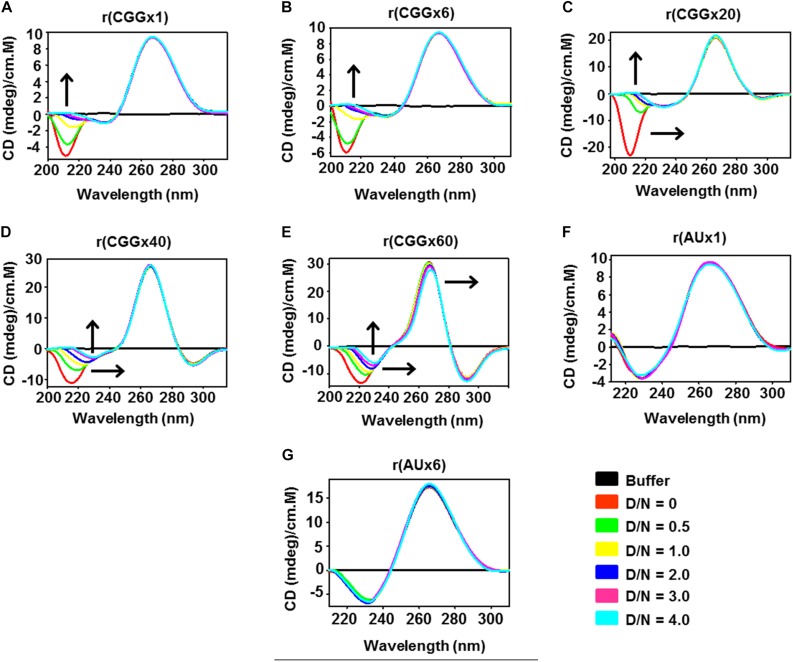
Circular Dichroism spectroscopy study of different CGG RNAs repeats and AU duplex RNA with increasing concentration of Curcumin. Plot **(A)** r(CGGx1), **(B)** r(CGGx6), **(C)** r(CGGx20), **(D)** r(CGGx40) and **(E)** r(CGGx60) shows spectral variation with the increasing concentration of Curcumin, whereas no significant changes were observed with **(F)** r(AUx1), and **(G)** r(AUx6) duplex RNAs. Arrow heads denotes the shifting of negative and positive peaks upon drug titration. D/N denotes drug by nucleotide ratio. Each spectrum was recorded three times and average changes were plotted.

Expanded CGG RNA repeats fold into RNA hairpins structure, which can be modified by small molecule ligands (Disney et al., 2012; Tran et al., 2014; Yang et al., 2016a). Thus, we investigated structural changes in CGG RNA in the absence and presence of Curcumin by Circular Dichroism (CD) spectroscopy (Figure 3). Typical CD spectra of r(CGG)^exp^ RNAs display positive absorption peak at 265–270 nm and a negative absorption peaks around 215–220 nm, which is characteristic of a A type RNA conformation as previously reported (Kypr et al., 2009).

The gradual addition of Curcumin to CGG RNAs solution causes spectral variations such as hypochromic shift (decrease in ellipticity) and red shift in negative peaks in a concentration dependent manner owing to the formation of Curcumin-RNA complex (Figures 3A–E). Of interest, spectral changes were nearly similar to all length of tested r(CGG) repeat RNAs, which highlights the similar binding geometry of Curcumin with all r(CGG) repeat RNAs (Figures 3A–E). Furthermore, constant hypochromic shift and red shift of CGG RNA are indicative of a π-π stacking interactions between Curcumin and CGG repeats RNA (Giri and Kumar, 2008; Das et al., 2011). As a control, no significant spectral changes were found in the CD spectra of r(AU) duplex paired RNAs (Figures 3F–G). Thus, CD analysis confirmed that Curcumin forms a complex with CGG repeat RNAs.

### CD Thermal Denaturation Study of CGG Repeats RNA With Curcumin

**FIGURE 4:**
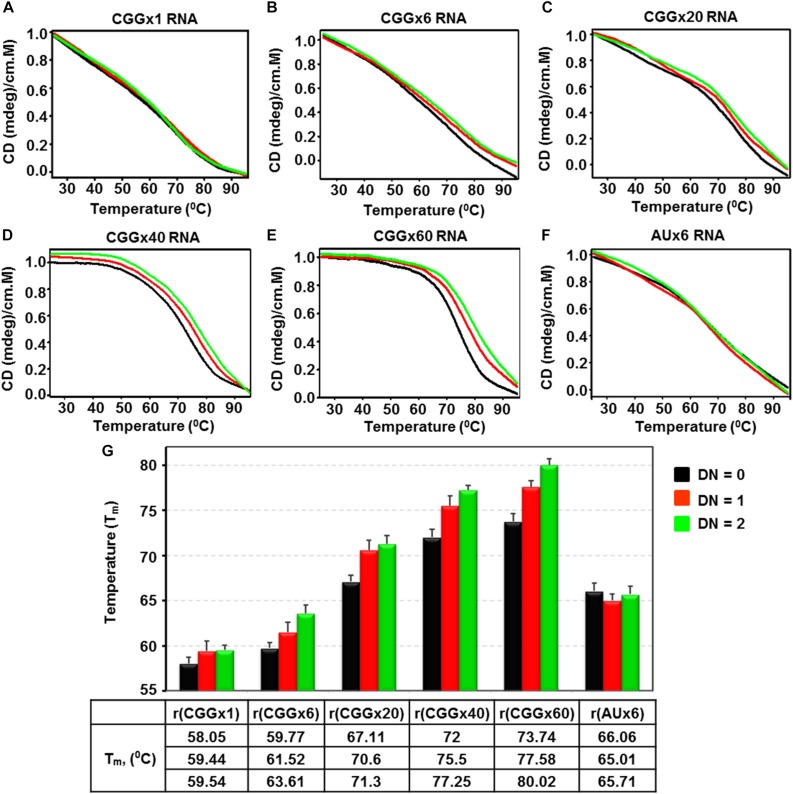
Diagram represents CD melting titration of r(CGG)n repeats containing RNAs and r(AU) fully paired RNA as a function of Curcumin concentration. Diagram shows titration plot of **(A)** r(CGGx1), **(B)** r(CGGx6), **(C)** r(CGGx20), **(D)** r(CGGx40), **(E)** r(CGGx60) and **(F)** r(AUx6) duplex RNA with increasing concentration of Curcumin. **(G)** Bar graph represents the melting temperature (T_m_) values of respective RNA at different drug by nucleotide ratio (D/N). All the melting titrations were repeated three times and the average values are presented.

To assess the effect of Curcumin on the thermal profile of CGG repeat RNAs, which could affect CGG associated splicing defects and RAN translation (Yang et al., 2016b), we used CD thermal denaturation assays. CGG RNA thermal profile were monitored at 267 nm and the changes in melting temperature (ΔT_*m*_) were of 1.49, 3.48, 4.19, 5.25, and 6.28°C for r(CGGx1), r(CGGx6), r(CGGx20), r(CGGx40), and r(CGGx60), respectively (Figures 4A–E). In contrast, no change in melting temperature was found as function of Curcumin concentration with AU pair duplex RNA (Figure 4F). Interestingly, large changes in melting temperature were found in RNAs with higher repeat numbers as compared to lower repeat RNAs (Figure 4G). In conclusion, Curcumin enhances the thermal stability of all tested CGG targeted RNAs, indicating that Curcumin stabilizes the CGG RNA structure, which may prevent ribosomal assembly to initiate RAN translation and/or interaction of the RNA binding proteins that mediate FXTAS-splicing defects.

### Electrophoretic Mobility Shift and PCR Inhibition of r(CGG)^exp^ With Curcumin

**FIGURE 5:**
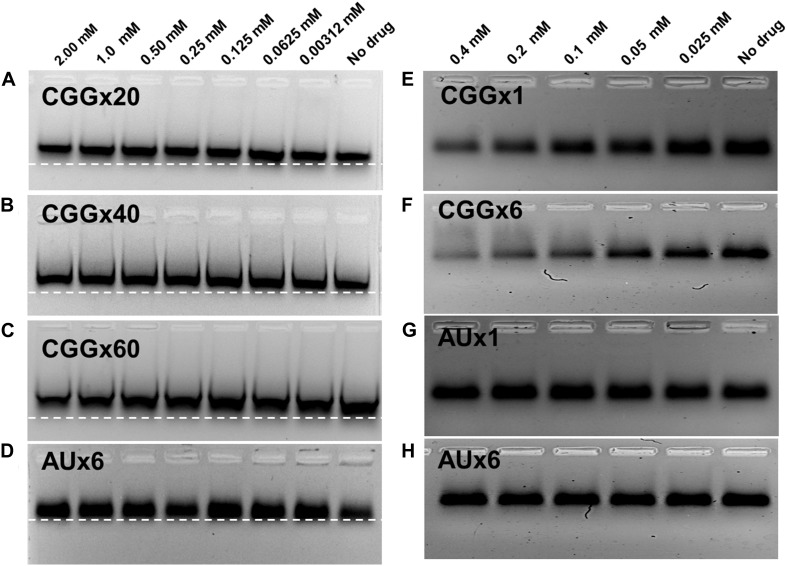
Electrophoretic mobility shift (EMSA), and PCR inhibition assay. EMSA plot shows significant retardation in mobility of CGG repeats RNAs with the increasing concentration of Curcumin. **(A)** r(CGGx20) **(B)** r(CGGx40) and **(C)** r(CGGx60) RNA. In contrast no significant retardation was detected with **(D)** r(AU)6× duplex RNA. PCR inhibition assay of CGG DNA templates were performed with Curcumin as a function of concentration; decrease in the PCR band intensity suggests the binding of Curcumin with CGG Repeat **(E)** (CGGx1) and **(F)** (CGGx6). In contrast, no significant inhibition was observed in the PCR band intensity of AU duplex DNA templates **G)** (AUx1) and **(H)** (AUx6).

For further validations of our ITC and fluorescence titration binding assays, we performed electrophoretic gel shift mobility and PCR stop assays. Gel shift assay was performed with 20 μM r(CGG)^exp^ RNAs incubated at RT for 30–60 min with a serial dilution of Curcumin and loaded on gels indicate retardation in migration for all r(CGG)^exp^ RNAs (Figures 5A–C and Supplementary Figures S4A–C). Of interest, higher repeat CGG RNAs such as r(CGGx40) and r(CGGx60) showed maximum retardation in migration (Figures 5B,C). In contrast, AU paired duplex RNA showed no retardation in migration which, confirm selective binding of Curcumin to r(CGG)^exp^ RNAs (Figure 5D and Supplementary Figure S4D). In parallel, we also tested Curcumin binding to CGG repeats by polymerase chain reaction (PCR) stop assays. This experiment is based on the hypothesis that if Curcumin binds to CGG repeats, it may impair the polymerase activity during extension. Consistent with this model, the successive addition of Curcumin decreases the band intensity of the PCR product (Figures 5E,F and Supplementary Figures S4E,F). As a control, no considerable changes in PCR band intensity were detected with AU paired DNA (Figures 5G,H and Supplementary Figures S4G,H). In addition, molecular docking experiments indicate that Curcumin binding to CGG repeats RNA involves both GxG loop (GxG-2 and GxG-5) with a −7.35 and −6.71 kcal/mol binding energy, respectively (Supplementary Figure S5; Kumar et al., 2011). These data confirm the interaction of Curcumin with RNAs containing expansion of CGG repeats.

### Curcumin Reverse Pre-mRNA Alternative Splicing Defects in FXTAS Model Cell Line

Next, we determined cellular potency of Curcumin to correct FXTAS-associated pre-mRNA alternative splicing defects in cultured cell using survival of motor neuron 2 (*SMN2)* and B-cell lymphoma × *(Bcl-x)* minigenes.

**FIGURE 6:**
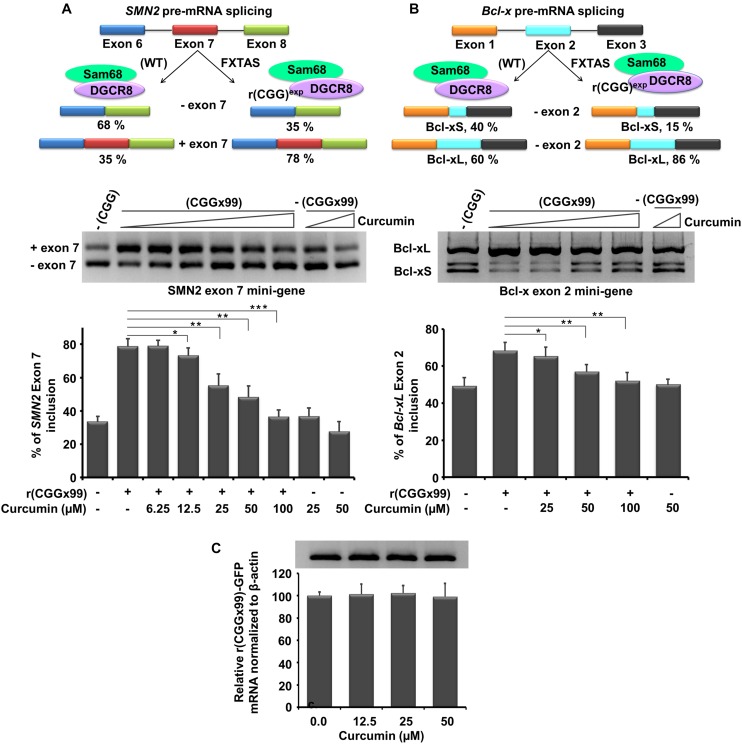
*In vitro* potency of Curcumin in FXTAS cultured cell line. Curcumin treatment shown was to ameliorate FXTAS-associated pre-mRNA alternative splicing dysregulation of *SMN2* and *Bcl-x* minigenes. Briefly, HEK cells were co-transfected *SMN2* and *Bcl-x* mini gene with the plasmid containing CGG repeat (CGGx99). *SMN2* splicing regulation is controlled by DGCR8 and Sam68 proteins. Upon sequestration and inactivation of these proteins the splicing of *SMN2* is dysregulated [**(A)** top, left]. FXTAS cellular model system treated with Curcumin, improvement of *SMN2* splicing defects were restored to near to wild type at 100 μM. Similar to *SMN2, Bcl-x* splicing is also controlled by DGCR8 and Sam68 and mis-splicing of *Bcl-x* caused due to presence of r(CGG)^exp^ were ameliorated with Curcumin treatment in FXTAS cell models [**(B)** top, right]. Importantly, Curcumin does not alter EGFP mRNA expression [**(C)** bottom]. Standard deviation represented by Error bars. Statistical significance were calculated by One-way ANOVA where **p < 0.01; *p < 0.05. Asterisk represent the statistical variation between control and treated.

Previous studies reported that Sam68, a splicing regulator, sequestered by expanded CGG repeat transcripts causes dysregulation of *SMN2* and *Bcl-x* pre-mRNA splicing (Sellier et al., 2010; Disney et al., 2012). In this study, r(CGG)×99 repeats containing plasmid and SMN2 minigene were cotransfected in HEK293 cells then increasing concentration of Curcumin were given in the growth media for 24 hrs (Supplementary Table S4). Approximately 35% *SMN2* mature mRNA contain exon 7 in absence r(CGG)×99, while in presence of r(CGG)×99 approximately 65% of exon 7 included in *SMN2* mRNA (Figure 6A; Disney et al., 2012). Notably, *SMN2* splicing defect improvement was observed at a concentration of 25 μM Curcumin. More significant improvement of splicing defects was observed at higher concentration. Curcumin significantly restored pre-mRNA alternative splicing defects near to wild type (lack CGGx99) pattern of *SMN2* at 100 μM.

In contrast, Curcumin does not influence splicing pattern of *SMN2* minigenes in the absence of CGGx99 expansion (Figure 6A). In addition, Bcl-x minigene showed two different splicing products *Bcl-xL* and *Bcl-xS* isoform. In CGG transfected cells, 68% of *Bcl-x* mRNA showed *Bcl-xL* isoform where as in normal untransfected cells, only 50% of *Bcl-xL* isoform is present (Disney et al., 2012). Curcumin improved mis-splicing defect of *Bcl-xL* in the concentration dependent fashion. Small improvements in mis-splicing defect of *Bcl-x*L minigene were observed at 25 μM (Figure 6B). Further, significant improvement of mis-splicing was detected at higher concentration (100 μM). However, it does not significantly alter the alternative splicing pattern of *Bcl-x* minigene with the plasmid lacking CGG expansion (Figure 6B).

In addition, control experiments were assessed to check the GFP mRNA expression in presence of Curcumin, till 50 μM concentration no considerable effect were detected (Figure 6C). Another control, cardiac troponin T (*cTNT*) minigene was used to investigate the alternative splicing, as their splicing pattern is not influenced by the CGG expansion (Disney et al., 2012). Interestingly, Curcumin treatment does not affect the alternative splicing of *cTNT* minigene (Supplementary Figure S6). Thus, our data suggested that Curcumin does not affect global splicing efficiency of genes that are not influenced by CGG expansion.

### Curcumin Reduces Toxic FMR1polyG Protein Inclusions

**FIGURE 7:**
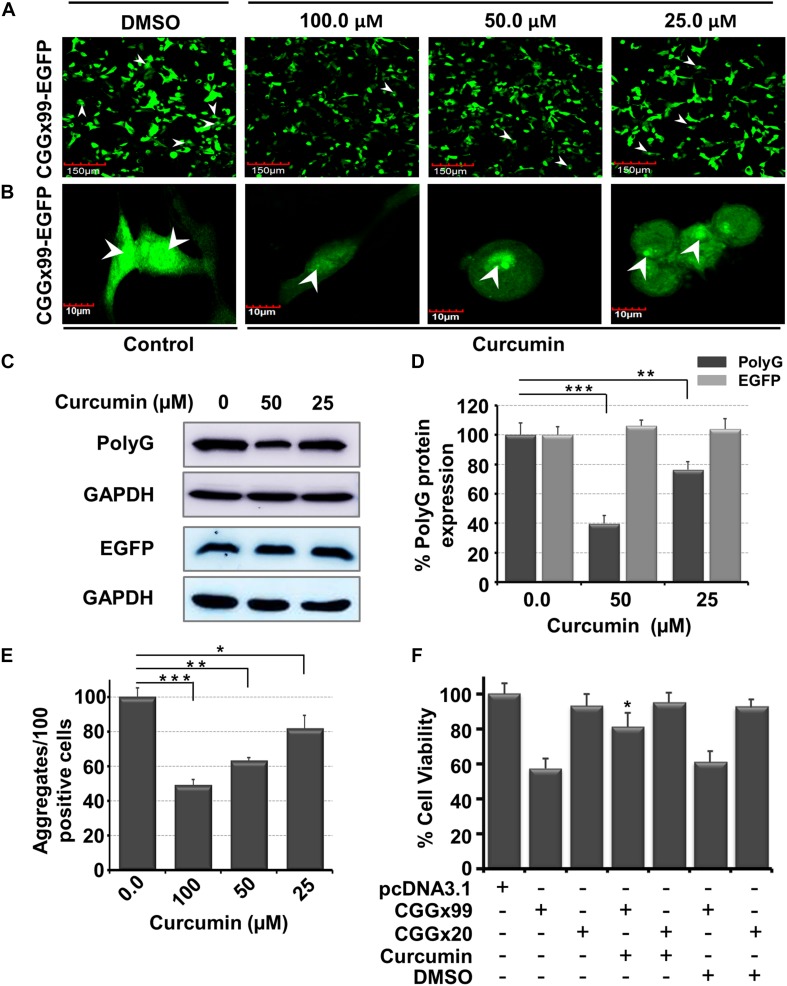
Diagram shows *in vitro* potency of Curcumin in FXTAS cultured cell models. In FXTAS, CGG expansion triggers the non-canonical protein translation leading to protein aggregate formation inside the cell. **(A,B)** Micrograph showing the effect of Curcumin treatment in transiently transfected [(CGGx99)-EGFP] HEK cell. **(A)** 20× image and **(B)** 60× image of DMSO (control) and Curcumin for 24 h. **(C)** Blot showing the reduction of polyG aggregates formation as a function of Curcumin concentration with control GAPDH and EGFP plasmid (lacking CGG repeat). **(D)** Bar graph showing the percentage reduction of polyG aggregate in presence of Curcumin. **(E)** Plot denotes Curcumin treatment reduces number of protein aggregates in the cell. **(F)** Bar graph showing improvement of cell viability of transfected HEK cells with plasmid CGGx20, CGGx99 and EGFP in presence of Curcumin. Standard deviation represented by Error bars. One-way ANOVA was used for determination of statistical significance except cell viability enhancement **(F)** assay where student *t*-test were utilized. ***p < 0.001; **p < 0.01; *p < 0.05.

In addition to correction of splicing defects, we also determined the potency of Curcumin to inhibit the pathogenic non-canonical translational of expanded CGG repeats. Expansions of CGG repeats in *FMR1* are translated into FMRpolyG, a small glycine-rich protein that form intranuclear inclusions, which is a another hallmark of FXTAS. HEK cells were transiently transfected with a CGGx99 repeats containing plasmid incorporated in the human 5′UTR of *FMR1* sequence and cloned in the glycine frame with the EGFP protein. This construct expresses the FMRpolyG protein from its natural ACG near-cognate start codon (Sellier et al., 2013). Previously small molecules have been reported, against different repeats containing mRNA such as r(GGGGCC)^exp^ and r(CUG)^exp^ that cause ALS/FTD and DM1 disease, respectively (Simone et al., 2018; Angelbello et al., 2019). A potent small molecule should interfere with the r(CGG)^exp^ hairpin structure that does not allow ribosomal machinery to initiate RAN translation and reduce protein mediated toxicity. Importantly, Curcumin showed a potent reduction in expression of the FMRpolyG proteins, approximately ∼25 and ∼60% of RAN translation was inhibited at 25 and 50 μM of Curcumin, respectively (Figures 7A–D). As a control, Curcumin does not modify translation of the GFP protein from a control plasmid lacking CGG repeats (Figures 7C,D and Supplementary Figure S7). In addition, protein aggregate quantifications were performed manually in Curcumin treated condition; approx. 40% and 50% of RAN translation were impaired at 50 and 100 μM, respectively (Figure 7E). Thus, enhancing thermostability of r(CGG)^exp^ through compounds correlates with the reduction of protein aggregates (Yang et al., 2015, 2016b).

FXTAS is a progressive neurological disorder typically characterized by neuronal cell loss. Thus, we tested whether Curcumin can prevent cell cytotoxicity caused by the expression of CGG expansion. Interestingly, Curcumin restores normal cell viability of HEK293 cells expressing CGGx99 repeats construct (Figure 7D). As controls, we assessed the cytotoxicity of Curcumin in HEK293 cells and normal fibroblasts (GM00357) as well as in FXTAS patient derived fibroblasts (GM04026), but found no overt cytotoxicity of Curcumin (Supplementary Figure S8). Furthermore, cellular intake of Curcumin was also assessed after 24 hrs in patient derived cells. Confocal microscopy images suggest that Curcumin can enter inside the cell (Supplementary Figure S9). Taken together these data suggested that Curcumin binds selectively to CGG repeats and ameliorate cell viability in FXTAS cell models without presenting overt toxicity.

## Discussion

It is well known that expansion of CGG repeats in the 5′ UTR of mutant FMR1 transcripts causes FXTAS (Hoem et al., 2011; Kong et al., 2017). Previously, it was reported that CGG repeat DNA and RNA sequences form 1 × 1 GG internal hairpin and tetraplex structure (Khateb et al., 2004; Zumwalt et al., 2007). It is extensively reported that CGG hairpin structure exerts toxic gain of function *via* sequestration of RNA binding proteins in RNA foci and cause splicing defects (Iwahashi et al., 2006; Sellier et al., 2010). CGG repeats also produces toxic polyglycine protein, FMRpolyG *via* repeat-associated non-AUG (RAN) translation (Todd et al., 2013; Sellier et al., 2017). Previous studies have shown that selective targeting of CGG hairpin structures *via* small molecules to reduce both RNA foci and PolyG aggregates formation is the most successful approach (Yang et al., 2015, 2016b). Thus, we sought to find out natural small molecule modulator that rescues r(CGG)^exp^ based toxicity. Here, we identified, a small molecule Curcumin that target CGG RNA motifs with high affinity using various biophysical methods. We also provide the evidence of significant improvement in the pre-mRNA alternative splicing defect of affected genes and reduces the number and level of polyG protein aggregates in r(CGGx99) developed cell model. Furthermore, Curcumin increases the survival of toxic CGG repeat containing plasmid transfected cells.

Initially, fluorescence based screening of Curcumin against different mismatched RNA motif and fully paired RNA showed high affinity of Curcumin with CGG RNA motif. Curcumin portrays varied binding characteristics with different mismatched RNA motifs as it has different binding constant values for different RNA sequences. Lower affinity of Curcumin with different higher repeat mismatched RNAs, yeast t-RNA and G-quadruplex forming DNA suggests the selectively of Curcumin with CGG expansion. In addition, increase in the number of CGG repeat enhances the binding affinity of Curcumin as revealed by dissociation constant difference of r(CGGx6) and r(CGGx60) RNA. Taken together, binding of Curcumin appear to be sequence, topology and stoichiometry dependent (Parkesh et al., 2012; Khan et al., 2018, 2019a).

Furthermore, ITC data supports the interactions between Curcumin with CGG expansion (Nafisi et al., 2009). In general, for the energetically favorable interactions between two molecules the enthalpy change (ΔH) should be negative (Falconer and Collins, 2011). Complementing with fluorescence result, ITC portrays similar binding pattern of Curcumin with CGG RNA over AUx6 duplex RNA. As the repeat length increases from 6 repeat to 40 and 60 repeats, binding affinity of Curcumin increased approximately by 50 folds. However, small enhancements in affinity were observed in between other higher (20/40/60) repeat RNAs. High dissociation constant values and exothermic peak concluded stronger interaction with intercalative mode of binding between Curcumin and CGG RNAs (Das et al., 2011).

Topological studies based on CD spectroscopy of CGG RNA with Curcumin depicted the significant changes till D/N = 4. It is interesting to note that, spectral changes pattern were nearly similar during titration of Curcumin with all r(CGG) repeat RNAs which further suggested the similar mode of binding. Moreover, constant CD spectrum changes of CGG RNA elucidated the intercalation mode binding of Curcumin within CGG hairpin structure (Das et al., 2011; Khan et al., 2018, 2019a). Similarly, in melting studies, the increase in the melting temperature in presence of Curcumin depicts the stabilization of the targeted RNA structures. Previous studies have been reported that stabilization of CGG repeat RNA *via* small molecules ameliorate FXTAS associated pathogenic defects (Yang et al., 2016b). Recently our group reported some small molecules that thermodynamically stabilize 5′CAG/3′GAC repeat RNA hairpin structure which prevent polyQ aggregation in Huntington disorder (Khan et al., 2019a, b). In continuation, significant reduction in mobility of CGG RNA in presence of Curcumin also signifies the strong binding. The shift in the mobility was due to the sequence and motif specific interactions that allow the formation of stable complex between Curcumin and r(CGG)^exp^ RNAs. Moreover PCR stop assay also suggested the same (Mishra et al., 2019).

Similarly, Curcumin mediated stabilization of CGG repeat RNA could improve splicing defect and RAN translation. As we observed, Curcumin treatment improves the splicing defect in developed cell model (Yang et al., 2016a). Curcumin could act through masking the sequestration of RNA regulatory protein to bind with the CGG RNA and allow them to perform their normal function. Improvement of splicing defects, as we observed is consistent with the reports that binding of small molecules to 5′CNG/3′GNC RNA motifs improve the splicing defect (Disney et al., 2012; Kumar et al., 2012; Parkesh et al., 2012; Tran et al., 2014). Here in our study, Curcumin rescue SMN2 and Bcl-x minigenes mis-splicing defect near to wild type without affecting normal regulation of same in the absence of CGG expansion.

In addition, the decrease in the formation of homopolymeric polyG aggregates or inhibition of RAN translation was observed in Curcumin treated cultured cells. There are two working mechanism by which compound could rescue RAN translation. First model, enhancing thermal stability of r(CGG)^exp^ through small molecule correlates with the inhibitory effect of RAN translation (Yang et al., 2015, 2016b; Khan et al., 2018) as stabilization impede ribosomal complex *read* through to the mRNA. However, there are many factors which may involve in RANT. Considering the fact, here in this study Curcumin increased the thermal stability of r(CGG)^exp^ motifs that is vital for the effective inhibition of RANT, further support the effect of RNA stabilization on RANT. Second model, binding of compound to CGG expansion interrupt ribosomal machinery to start translation from random position. Additionally, Curcumin have been reported for neuroprotective effect in other neurological disorder also such as Alzheimer’s, Parkinson’s, frontotemporal dementia, and Huntington disease etc. Administration of Curcumin improved the cell viability through various mechanism including inhibits amyloid formation and its toxicity in Alzheimer disease, reduce oxidative damage, inflammation, and synuclein protein aggregation in Parkinson disease, decrease aggregation of tau proteins in frontotemporal dementia. Curcumin treatment attenuates ischemia-induced oxidative stress and neuronal cell death in hippocampal tissue. Further, Curcumin inhibits the accumulation and aggregation of prion protein inside the neuroblastoma cell. It also facilitates anti-anxiety activity by decrease level of TNF-α and MMP-9 in Autism spectrum disorder (Cole et al., 2007; Bhat et al., 2019).

## Conclusion

There is a strong need of effective and safe therapeutic modalities for FXTAS. Previous studies have reported that the stabilization of CGG repeat RNA *via* small molecule ameliorates FXTAS associated pathogenic defects (Yang et al., 2016b). Recently, our group has published few small molecules that thermodynamically stabilize expanded CAG RNA hairpin motifs which prevent polyQ aggregation in Huntington disorder (Khan et al., 2018, 2019a). Herein, we have demonstrated that Curcumin binds to r(CGG)^exp^ and potently reverses FXTAS-associated pathogenic defects. Biophysical characterization using CD spectroscopy, CD melting and gel retardation illustrate the specific binding of Curcumin with CGG repeats RNA. Importantly, Curcumin improves pre-mRNA *SMN2* and *Bcl-x* alternative splicing defect caused by expression of CGG expansions. Similarly, Curcumin also prevents non-canonical translation of the CGG repeating RNA into the toxic inclusion of FMRpolyG protein. Curcumin may inhibit translation through two potential mechanisms. First, it has been reported that longer CGG expansions (thermodynamic more stable) or small molecule enhancing thermostability of r(CGG)^exp^ correlates with their inhibitory effect on RAN translation (Yang et al., 2015; Verma et al., 2019b) as RNA hairpin stabilization impedes ribosomal complex *read* through to mRNA. A second model is that binding of compounds to CGG expansions can simply occupy the RNA and prevent ribosomal machinery to start non-canonical translation. Similarly, Curcumin binding to CGG repeats can prevent further association and interaction of RNA binding proteins that leads to FXTAS-associated splicing defects. Furthermore, Curcumin is able to cross blood brain barrier due to its lipophilic nature, despite of its poor bioavailability. Henceforth, Curcumin reach brain in biological effective concentration and ameliorate neurodegeneration (Begum et al., 2008; Schiborr et al., 2010). To further increase the bioavailability of Curcumin, researchers have been designed different nanoparticles (Nanocurcumin) to enhance its pharmacological activity (Barbara et al., 2017; Dende et al., 2017). In conclusion, our data provide evidence that Curcumin could be used for the therapeutics of CGG associated diseases FXTAS and FXPOI. However, further animal models studies are required to reach any conclusion.

## Data Availability Statement

All datasets generated for this study are included in the article/Supplementary Material.

## Author Contributions

AK conceived and designed the project. AV, EK, and SM performed the experiments. AV and AK analyzed the data and drafted the manuscript. AK, AM, and NC-B performed critical analysis to the manuscript.

## Conflict of Interest

The authors declare that the research was conducted in the absence of any commercial or financial relationships that could be construed as a potential conflict of interest.

## References

[B1] Angelbello A. J. Rzuczek S. G. Mckee K. K. Chen J. L. Olafson H. Cameron M. D. (2019). Precise small-molecule cleavage of an r(CUG) repeat expansion in a myotonic dystrophy mouse model. *Proc. Natl. Acad. Sci. U.S.A.* 116 7799–7804. 10.1073/pnas.1901484116 30926669 PMC6475439

[B2] Balendra R. Isaacs A. M. (2018). *C9orf72*-mediated ALS and FTD: multiple pathways to disease. *Nat. Rev. Neurol.* 14 544–558. 10.1038/s41582-018-0047-2 30120348 PMC6417666

[B3] Barbara R. Belletti D. Pederzoli F. Masoni M. Keller J. Ballestrazzi A. (2017). Novel curcumin loaded nanoparticles engineered for blood-brain barrier crossing and able to disrupt Abeta aggregates. *Int. J. Pharm.* 526 413–424. 10.1016/j.ijpharm.2017.05.015 28495580

[B4] Begum A. N. Jones M. R. Lim G. P. Morihara T. Kim P. Heath D. D. (2008). Curcumin structure-function, bioavailability, and efficacy in models of neuroinflammation and Alzheimer’s disease. *J. Pharmacol. Exp. Ther.* 326 196–208. 10.1124/jpet.108.137455 18417733 PMC2527621

[B5] Berry-Kravis E. Abrams L. Coffey S. M. Hall D. A. Greco C. Gane L. W. (2007). Fragile X-associated tremor/ataxia syndrome: clinical features, genetics, and testing guidelines. *Mov. Disord.* 22 2018–2030, quiz 2140. 17618523 10.1002/mds.21493

[B6] Bhat A. Mahalakshmi A. M. Ray B. Tuladhar S. Hediyal T. A. Manthiannem E. (2019). Benefits of curcumin in brain disorders. *Biofactors* 45 666–689. 10.1002/biof.1533 31185140

[B7] Buijsen R. A. Sellier C. Severijnen L.-A. Oulad-Abdelghani M. Verhagen R. F. M. Berman R. F. (2014). FMRpolyG-positive inclusions in CNS and non-CNS organs of a fragile X premutation carrier with fragile X-associated tremor/ataxia syndrome. *Acta Neuropathol. Commun.* 2:162. 10.1186/s40478-014-0162-2 PMC4254384 25471011

[B8] Bushara K. Bower M. Liu J. Mcfarland K. N. Landrian I. Hutter D. (2013). Expansion of the spinocerebellar ataxia type 10 (SCA10) repeat in a patient with Sioux native American ancestry. *PLoS One* 8:e81342. 10.1371/journal.pone.0081342 24278426 PMC3835687

[B9] Calin G. A. Croce C. M. (2006). MicroRNAs and chromosomal abnormalities in cancer cells. *Oncogene* 25 6202–6210. 10.1038/sj.onc.1209910 17028600

[B10] Cleary J. D. Pattamatta A. Ranum L. P. W. (2018). Repeat-associated non-ATG (RAN) translation. *J. Biol. Chem.* 293 16127–16141. 10.1074/jbc.R118.003237 30213863 PMC6200949

[B11] Coffey S. M. Cook K. Tartaglia N. Tassone F. Nguyen D. V. Pan R. (2008). Expanded clinical phenotype of women with the FMR1 premutation. *Am. J. Med. Genet. Part A* 146A 1009–1016. 10.1002/ajmg.a.32060 18348275 PMC2888464

[B12] Cole G. M. Teter B. Frautschy S. A. (2007). Neuroprotective effects of curcumin. *Adv. Exp. Med. Biol.* 595 197–212. 10.1007/978-0-387-46401-5_8 17569212 PMC2527619

[B13] Das A. Bhadra K. Suresh Kumar G. (2011). Targeting RNA by small molecules: comparative structural and thermodynamic aspects of aristololactam-beta-D-glucoside and daunomycin binding to tRNA(phe). *PLoS One* 6:e23186. 10.1371/journal.pone.0023186 21858023 PMC3156712

[B14] Dende C. Meena J. Nagarajan P. Nagaraj V. A. Panda A. K. Padmanaban G. (2017). Nanocurcumin is superior to native curcumin in preventing degenerative changes in experimental cerebral malaria. *Sci. Rep.* 7:10062. 10.1038/s41598-017-10672-9 28855623 PMC5577147

[B15] Disney M. D. Liu B. Yang W. Y. Sellier C. Tran T. Charlet-Berguerand N. (2012). A small molecule that targets r(CGG)(exp) and improves defects in fragile X-associated tremor ataxia syndrome. *ACS Chem. Biol.* 7 1711–1718. 10.1021/cb300135h 22948243 PMC3477254

[B16] Falconer R. J. Collins B. M. (2011). Survey of the year 2009: applications of isothermal titration calorimetry. *J. Mol. Recognit.* 24 1–16. 10.1002/jmr.1073 21157775

[B17] Fraint A. Vittal P. Szewka A. Bernard B. Berry-Kravis E. Hall D. A. (2014). New observations in the fragile X-associated tremor/ataxia syndrome (FXTAS) phenotype. *Front. Genet.* 5:365. 10.3389/fgene.2014.00365 25368631 PMC4201107

[B18] Freibaum B. D. Taylor J. P. (2017). The role of dipeptide repeats in *C9ORF72*-related ALS-FTD. *Front. Mol. Neurosci.* 10:35. 10.3389/fnmol.2017.00035 28243191 PMC5303742

[B19] Freyer M. W. Lewis E. A. (2008). Isothermal titration calorimetry: experimental design, data analysis, and probing macromolecule/ligand binding and kinetic interactions. *Methods Cell Biol.* 84 79–113. 10.1016/s0091-679x(07)84004-0 17964929

[B20] Giri P. Kumar G. S. (2008). Self-structure induction in single stranded poly(A) by small molecules: studies on DNA intercalators, partial intercalators and groove binding molecules. *Arch. Biochem. Biophys.* 474 183–192. 10.1016/j.abb.2008.03.013 18387354

[B21] Green K. M. Linsalata A. E. Todd P. K. (2016). RAN translation-what makes it run? *Brain Res.* 1647 30–42. 10.1016/j.brainres.2016.04.003 27060770 PMC5003667

[B22] Groh M. Lufino M. M. P. Wade-Martins R. Gromak N. (2014). R-loops associated with triplet repeat expansions promote gene silencing in Friedreich ataxia and fragile X syndrome. *PLoS Genet.* 10:e1004318. 10.1371/journal.pgen.1004318 24787137 PMC4006715

[B23] Hagerman P. J. Hagerman R. J. (2015). Fragile X-associated tremor/ataxia syndrome. *Ann. N. Y. Acad. Sci.* 1338 58–70. 10.1111/nyas.12693 25622649 PMC4363162

[B24] Hagerman R. J. Hagerman P. (2016). Fragile X-associated tremor/ataxia syndrome - features, mechanisms and management. *Nat. Rev. Neurol.* 12 403–412. 10.1038/nrneurol.2016.82 27340021

[B25] He F. Krans A. Freibaum B. D. Taylor J. P. Todd P. K. (2014). TDP-43 suppresses CGG repeat-induced neurotoxicity through interactions with HnRNP A2/B1. *Hum. Mol. Genet.* 23 5036–5051. 10.1093/hmg/ddu216 24920338 PMC4159148

[B26] Hoem G. Raske C. R. Garcia-Arocena D. Tassone F. Sanchez E. Ludwig A. L. (2011). CGG-repeat length threshold for FMR1 RNA pathogenesis in a cellular model for FXTAS. *Hum. Mol. Genet.* 20 2161–2170. 10.1093/hmg/ddr101 21389081 PMC3090194

[B27] Iwahashi C. K. Yasui D. H. An H. J. Greco C. M. Tassone F. Nannen K. (2006). Protein composition of the intranuclear inclusions of FXTAS. *Brain* 129 256–271. 10.1093/brain/awh650 16246864

[B28] Kearse M. G. Green K. M. Krans A. Rodriguez C. M. Linsalata A. E. Goldstrohm A. C. (2016). CGG repeat-associated non-AUG translation utilizes a cap-dependent scanning mechanism of initiation to produce toxic proteins. *Mol. Cell* 62 314–322. 10.1016/j.molcel.2016.02.034 27041225 PMC4854189

[B29] Khan E. Biswas S. Mishra S. K. Mishra R. Samanta S. Mishra A. (2019a). Rationally designed small molecules targeting toxic CAG repeat RNA that causes Huntington’s disease (HD) and spinocerebellar ataxia (SCAs). *Biochimie* 163 21–32. 10.1016/j.biochi.2019.05.001 31075282

[B30] Khan E. Mishra S. K. Mishra R. Mishra A. Kumar A. (2019b). Discovery of a potent small molecule inhibiting Huntington’s disease (HD) pathogenesis via targeting CAG repeats RNA and Poly Q protein. *Sci. Rep.* 9:16872. 10.1038/s41598-019-53410-z PMC6856162 31728006

[B31] Khan E. Tawani A. Mishra S. K. Verma A. K. Upadhyay A. Kumar M. (2018). Myricetin reduces toxic level of CAG repeats RNA in Huntington’s disease (HD) and Spino cerebellar ataxia (SCAs). *ACS Chem. Biol.* 13 180–188. 10.1021/acschembio.7b00699 29172480

[B32] Khateb S. Weisman-Shomer P. Hershco I. Loeb L. A. Fry M. (2004). Destabilization of tetraplex structures of the fragile X repeat sequence (CGG)n is mediated by homolog-conserved domains in three members of the hnRNP family. *Nucleic Acids Res.* 32 4145–4154. 10.1093/nar/gkh745 15302914 PMC514371

[B33] Kong H. E. Zhao J. Xu S. Jin P. Jin Y. (2017). Fragile X-associated tremor/ataxia syndrome: from molecular pathogenesis to development of therapeutics. *Front. Cell. Neurosci.* 11:128. 10.3389/fncel.2017.00128 28529475 PMC5418347

[B34] Kulkarni S. K. Dhir A. (2010). An overview of curcumin in neurological disorders. *Indian J. Pharm. Sci.* 72 149–154. 10.4103/0250-474X.65012 20838516 PMC2929771

[B35] Kumar A. Fang P. Park H. Guo M. Nettles K. W. Disney M. D. (2011). A crystal structure of a model of the repeating r(CGG) transcript found in fragile × syndrome. *Chembiochem* 12 2140–2142. 10.1002/cbic.201100337 21766409 PMC3379549

[B36] Kumar A. Parkesh R. Sznajder L. J. Childs-Disney J. L. Sobczak K. Disney M. D. (2012). Chemical correction of pre-mRNA splicing defects associated with sequestration of muscleblind-like 1 protein by expanded r(CAG)-containing transcripts. *ACS Chem. Biol.* 7 496–505. 10.1021/cb200413a 22252896 PMC3306454

[B37] Kypr J. Kejnovská I. Renciuk D. Vorlícková M. (2009). Circular dichroism and conformational polymorphism of DNA. *Nucleic Acids Res.* 37 1713–1725. 10.1093/nar/gkp026 19190094 PMC2665218

[B38] López-Morató M. Brook J. D. Wojciechowska M. (2018). Small molecules which improve pathogenesis of myotonic dystrophy type 1. *Front. Neurol.* 9:349. 10.3389/fneur.2018.00349 29867749 PMC5968088

[B39] Mishra S. K. Jain N. Shankar U. Tawani A. Sharma T. K. Kumar A. (2019). Characterization of highly conserved G-quadruplex motifs as potential drug targets in *Streptococcus pneumoniae*. *Sci. Rep.* 9:1791. 10.1038/s41598-018-38400-x 30741996 PMC6370756

[B40] Nafisi S. Adelzadeh M. Norouzi Z. Sarbolouki M. N. (2009). Curcumin binding to DNA and RNA. *DNA Cell Biol.* 28 201–208. 10.1089/dna.2008.0840 19364279

[B41] Nageshwaran S. Festenstein R. (2015). Epigenetics and triplet-repeat neurological diseases. *Front. Neurol.* 6:262. 10.3389/fneur.2015.00262 26733936 PMC4685448

[B42] Orr H. T. Zoghbi H. Y. (2007). Trinucleotide repeat disorders. *Annu. Rev. Neurosci.* 30 575–621. 17417937 10.1146/annurev.neuro.29.051605.113042

[B43] Parkesh R. Childs-Disney J. L. Nakamori M. Kumar A. Wang E. Wang T. (2012). Design of a bioactive small molecule that targets the myotonic dystrophy type 1 RNA via an RNA motif-ligand database and chemical similarity searching. *J. Am. Chem. Soc.* 134 4731–4742. 10.1021/ja210088v 22300544 PMC3306011

[B44] Pearson C. E. (2011). Repeat associated non-ATG translation initiation: one DNA, two transcripts, seven reading frames, potentially nine toxic entities! *PLoS Genet.* 7:e1002018. 10.1371/journal.pgen.1002018 21423665 PMC3053344

[B45] Schiborr C. Eckert G. P. Rimbach G. Frank J. (2010). A validated method for the quantification of curcumin in plasma and brain tissue by fast narrow-bore high-performance liquid chromatography with fluorescence detection. *Anal. Bioanal. Chem.* 397 1917–1925. 10.1007/s00216-010-3719-3 20419505

[B46] Sellier C. Buijsen R. A. M. He F. Natla S. Jung L. Tropel P. (2017). Translation of expanded CGG repeats into FMRpolyG is pathogenic and may contribute to fragile X tremor ataxia syndrome. *Neuron* 93 331–347. 10.1016/j.neuron.2016.12.016 28065649 PMC5263258

[B47] Sellier C. Freyermuth F. Tabet R. Tran T. He F. Ruffenach F. (2013). Sequestration of DROSHA and DGCR8 by expanded CGG RNA repeats alters microRNA processing in fragile X-associated tremor/ataxia syndrome. *Cell Rep.* 3 869–880. 10.1016/j.celrep.2013.02.004 23478018 PMC3639429

[B48] Sellier C. Rau F. Liu Y. Tassone F. Hukema R. K. Gattoni R. (2010). Sam68 sequestration and partial loss of function are associated with splicing alterations in FXTAS patients. *EMBO J.* 29 1248–1261. 10.1038/emboj.2010.21 20186122 PMC2857464

[B49] Sharp P. A. (2009). The centrality of RNA. *Cell* 136 577–580. 10.1016/j.cell.2009.02.007 19239877

[B50] Simone R. Balendra R. Moens T. G. Preza E. Wilson K. M. Heslegrave A. (2018). G-quadruplex-binding small molecules ameliorate *C9orf72* FTD/ALS pathology *in vitro* and *in vivo*. *EMBO Mol. Med.* 10 22–31. 10.15252/emmm.201707850 29113975 PMC5760849

[B51] Sofola O. A. Jin P. Qin Y. Duan R. Liu H. De Haro M. (2007). RNA-binding proteins hnRNP A2/B1 and CUGBP1 suppress fragile X CGG premutation repeat-induced neurodegeneration in a *Drosophila* model of FXTAS. *Neuron* 55 565–571. 10.1016/j.neuron.2007.07.021 17698010 PMC2215388

[B52] Todd P. K. Oh S. Y. Krans A. He F. Sellier C. Frazer M. (2013). CGG repeat-associated translation mediates neurodegeneration in fragile X tremor ataxia syndrome. *Neuron* 78 440–455. 10.1016/j.neuron.2013.03.026 23602499 PMC3831531

[B53] Tran T. Childs-Disney J. L. Liu B. Guan L. Rzuczek S. Disney M. D. (2014). Targeting the r(CGG) repeats that cause FXTAS with modularly assembled small molecules and oligonucleotides. *ACS Chem. Biol.* 9 904–912. 10.1021/cb400875u 24506227 PMC4287843

[B54] Urbanek M. O. Fiszer A. Krzyzosiak W. J. (2017). Reduction of Huntington’s disease RNA foci by CAG repeat-targeting reagents. *Front. Cell. Neurosci.* 11:82. 10.3389/fncel.2017.00082 28400719 PMC5368221

[B55] Verma A. K. Khan E. Bhagwat S. R. Kumar A. (2019a). Exploring the potential of small molecule-based therapeutic approaches for targeting trinucleotide repeat disorders. *Mol. Neurobiol.* 57 566–584. 10.1007/s12035-019-01724-4 31399954

[B56] Verma A. K. Khan E. Mishra S. K. Jain N. Kumar A. (2019b). Piperine modulates protein mediated toxicity in fragile X-associated tremor/ataxia syndrome through interacting expanded CGG repeat (r(CGG)(exp)) RNA. *ACS Chem. Neurosci.* 10 3778–3788. 10.1021/acschemneuro.9b00282 31264835

[B57] Warner K. D. Hajdin C. E. Weeks K. M. (2018). Principles for targeting RNA with drug-like small molecules. *Nat. Rev. Drug Discov.* 17 547–558. 10.1038/nrd.2018.93 29977051 PMC6420209

[B58] Wilton S. D. Fletcher S. (2005). RNA splicing manipulation: strategies to modify gene expression for a variety of therapeutic outcomes. *Curr. Gene Ther.* 5 467–483. 10.2174/156652305774329249 16250888

[B59] Yang W.-Y. Gao R. Southern M. Sarkar P. S. Disney M. D. (2016a). Design of a bioactive small molecule that targets r(AUUCU) repeats in spinocerebellar ataxia 10. *Nat. Commun.* 7:11647. 10.1038/ncomms11647 27248057 PMC4895354

[B60] Yang W. Y. He F. Strack R. L. Oh S. Y. Frazer M. Jaffrey S. R. (2016b). Small molecule recognition and tools to study modulation of r(CGG)(exp) in fragile X-associated tremor ataxia syndrome. *ACS Chem. Biol.* 11 2456–2465. 10.1021/acschembio.6b00147 27276216 PMC5549791

[B61] Yang W. Y. Wilson H. D. Velagapudi S. P. Disney M. D. (2015). Inhibition of non-ATG translational events in cells via covalent small molecules targeting RNA. *J. Am. Chem. Soc.* 137 5336–5345. 10.1021/ja507448y 25825793 PMC4856029

[B62] Zu T. Gibbens B. Doty N. S. Gomes-Pereira M. Huguet A. Stone M. D. (2011). Non-ATG–initiated translation directed by microsatellite expansions. *Proc. Natl. Acad. Sci. U.S.A.* 108 260–265. 10.1073/pnas.1013343108 21173221 PMC3017129

[B63] Zumwalt M. Ludwig A. Hagerman P. J. Dieckmann T. (2007). Secondary structure and dynamics of the r(CGG) repeat in the mRNA of the fragile X mental retardation 1 (FMR1) gene. *RNA Biol.* 4 93–100. 10.4161/rna.4.2.5039 17962727

