# Supplementary material for: Curcumin Regulates the r(CGG)exp RNA Hairpin Structure and Ameliorate Defects in Fragile X-Associated Tremor Ataxia Syndrome
Source: Front Neurosci. 2020 Apr 7;14:295. doi: 10.3389/fnins.2020.00295 (PMC7155420; doi:10.3389/fnins.2020.00295)
Supplement: Supplementary file 1 [file Data_Sheet_1.PDF]

Supporting information

Curcumin regulates the $r(CGG)^{exp}$ RNA hairpin structure and ameliorate defects in fragile X-associated tremor ataxia syndrome

Arun Kumar Verma[$], Eshan Khan[$], Subodh Kumar Mishra[$], Amit Mishra†, Nicolas Charlet-Berguerand‡ and Amit Kumar[$]*

[$]Discipline of Biosciences and Biomedical Engineering, Indian Institute of Technology Indore, Simrol, Indore 453552, India

†Cellular and Molecular Neurobiology Unit, Indian Institute of Technology Jodhpur, Rajasthan 342011, India

‡Translational Medicine and Neurogenetics, Institut de Génétique et de Biologie Moléculaire et Cellulaire (IGBMC), INSERM U964, CNRS UMR7104, University of Strasbourg, 1 Rue Laurent Fries, 67400 Illkirch-Graffenstaden, France

***Correspondence:** Amit Kumar, Associate Professor, Discipline of Biosciences and Biomedical Engineering, Indian Institute of Technology Indore, Simrol, Indore 453552, India Tel: +91-731-2438-771; Fax: +91-731-2438-721; Email: amitk@iiti.ac.in; amitkdbs@gmail.com

Table of Contents		
Material & Methods		S3-S9
Method References		S9
Tables S1-S4		S10-S14
Table S1	The dissociation constant (Kd) values as measured from fluorescence titration assay of Curcumin with different 1x1 single repeat RNA motif and AU duplex RNA control.	S10
Table S2	The dissociation constant (Kd) values as assessed from fluorescence titration assay of Curcumin with r(CGG)n RNAs and AU duplex RNAs & different DNA controls.	S11
Table S3	Thermodynamic and energy parameters as assessed from isothermal titration calorimetry of r(CGG)n repeat containing RNAs and AU duplex RNA control with Curcumin.	S12
Table S4	Semi quantitative PCR oligonucleotide sequences.	S13
Figure S1-S7		S14-S22
Figure S1	Diagram represent secondary structure of different 1x1 internal nucleotide loop (**5'CNG/3'CNG**) of RNAs sequences.	S14
Figure S2	Fluorescence titration assays plot of Curcumin with different single repeat 1x1 internal nucleotide RNA motif (**5'CNG/3'CNG**).	S15
Figure S3	Fluorescence titration assays plots of Curcumin with different r(CGG)n repeat containing RNAs motifs and different RNA & DNA controls.	S16
Figure S4	Quantification of mobility reduction of CGG repeats RNAs and PCR amplification inhibition in presence of Curcumin.	S17
Figure S5	Molecular docking study of CGGx3 repeats RNA with Curcumin.	S18
Figure S6	Representative semi RT-PCR gel image and quantification of CTNT mini-gene.	S19
Figure S7	Gel image and quantification of control GFP protein expression level as a function of Curcumin concentration.	S20
Figure S8	Determination of percentage viability of different cell line in presence of Curcumin.	S21
Figure S9	Image showing the cellular intake of Curcumin in pateint derived	S22

	cells.	

Material and Methods

Reagents

Curcumin molecule (catalogue no - 08511) and other reagent used for the buffer preparation such as EDTA, Tris-Hcl, NaOH, KCl, NaCl, Na_2HPO_4, NaH_2PO_4, K_2HPO_4 and KH_2PO_4 were procured from Merck and Co. Pvt. Ltd. The Curcumin molecule was used without further purification. The solvent reagent HPLC grade such as 2-butanol, ethanol, methanol, dimethyl sulfoxide (DMSO), deuterium oxide, deuterated DMSO were procured from sigma Aldrich. Midiprep plasmid isolation kit purchased from Invitrogen. All the PCR reagent including Taq polymerase, dNTP mix, primers, DNA template ($d(CGG)_{1-6}$ repeats) were also obtained from sigma Aldrich. Invitro transcription kit (MEGAscript® T7 Kit) was purchased from Thermo Fisher Scientific pvt. Ltd. For CGG hairpin motif formation, CGG repeat RNAs were prepared in 10mM phosphate buffer (pH 7.0) with 50 mM KCl. CGG RNAs heated for annealing by at 90 °C for 5 - 10 mins, and then allowed to cool down at the room temperature for 1-2 hrs. All the biophysical experiment was carried out using above mentioned buffer composition otherwise mentioned separately. For the cell culture experiment, Fetal bovine serum (FBS), culture media (Dulbecco's Modified Eagle Medium (DMEM), Antibiotic solution (ABAM), Phosphate buffer saline (PBS), were procured from Himedia. The Human Kidney cell line (HEK293) was procured from NCCS, Pune, India.

RNA Preparation and Purification

The synthetic DNA templates for invitro transcription (RNA preparation) were either amplified by PCR or/and cloned in a plasmid. Then they were used to prepare the RNA sequences by run-off transcription method using T7 RNA polymerase enzyme. The transcribed products were then purified by running in a 15% denaturing PAGE. The RNA band was visualized by UV torch and the RNA band was excised out and extracted with the help of 0.3M NaCl by tumbling down for overnight at 4 °C. The RNA was then ethanol precipitated for further purification.

Fluorescence based binding assay

The excitation and emission wavelength of Curcumin was calculated using Synergy™ H1 multi-mode microplate reader, which were further used to perform the Fluorescence titration assays. The target RNA sequences include CGG repeat RNAs along with the control sequences which included RNA AU duplex pair, yeast t-RNA and different DNAs like CT-DNA, c-Myc DNA, Bcl2 DNA were heated in the 10mM phosphate buffer at 90°C for 5-10mins and then allowed cooled down to RT before each set of experiment. A constant concentration of ligand in 10mM phosphate buffer was added to each of the twelve wells of the black corning 96 well plate and the target RNA/DNA solution was titrated keeping the last well blank. The fluorescence change on addition of the RNA/DNA was measured at the particular excitation and emission of the Curcumin with the help of the microplate reader at 25 °C. The obtained data were then analyzed using Sigma Plot 12.0 software (Systat Software, Chicago, USA).

$$\mathrm{f} = \frac{\mathrm{B}max^1 \times \mathrm{abs(x)}}{\mathrm{k_d}1 \times \mathrm{abs(x)}} + \frac{\mathrm{B}max^2 \times \mathrm{abs(x)}}{\mathrm{k_d}2 \times \mathrm{abs(x)}}$$

B_{max} = maximum number of binding sites. K_d = equilibrium binding constant

Isothermal titration calorimetry experiment

The working solution of the RNA were prepared by dissolving it in the 10mM potassium phosphate buffer and then properly annealed by heating it at 90°C for 5-10mins followed by slow cooling at room temperature (RT). The RNA sample was loaded into the cell while the working stock of the Curcumin was prepared in the same buffer and loaded to the syringe of the ITC instrument. Both the RNA as well as the ligand solutions was degassed before loading into the instrument to avoid any bubble. The ITC experiments were executed using the MicroCal™ isothermal titration calorimeter iTC200 instrument (GE healthcare) at a constant temperature of 298K where the Curcumin solution was injected to the RNA solution containing cell for a particular number if injections. A constant stirring speed of 750 rpm was maintained throughout the experiment. The binding data obtained due to ligand-RNA interaction was plotted using the MicroCal Origin software. The binding thermogram provided the dissociation constant (Kd) and other thermodynamic parameters obtained due to the ligand-RNA interaction.

Electrophoretic mobility shift assay

For each set of experiment, the 20 μM of each RNA samples were heated at 95°C in 10mM potassium phosphate buffer and cooled slowly to RT. Then the Curcumin was added at the optimum concentration and then serially diluted to rest of the tubes keeping the last well as blank. The Curcumin was incubated for 15 mins and then the 6X orange dye was mixed to the samples for loading into the gel. All samples were analyzed on a 3% agarose gel and run in 1X Tris-Borate-EDTA (TBE) buffer. The gels were pre-stained with ethidium bromide and visualized using analyzed on ImageQuant LAS 4000 (GE Healthcare).

PCR inhibition assay

The template for GGx1 (5'-GGAGAGGGUUUAAUCGGUACGAAAGUACGGAUUGGAU CCGCAAGG-3'), GGx6 (5'-GGAGAGGGUUUAAUCGGCGGCGGCGGCGGCGGUACG AAAGUACGGCGGCGGCGGCGGCGGAUUGGAUCCGCAAGG-3') and the complementary sequence (GGCCGGATCCTAAGATACGACTCACTATAGGGAGAGGG TTTAAT) was added to PCR mastermix containing 1X PCR buffer, 4.25mM MgCl2, 0.15 mM dNTPs and 2.5 units Taq DNA polymerase. The 25μl of mastermix was distributed in each of the six PCR tubes for each set of experiment and the Curcumin was added in the optimum concentration in the first tube and serially diluted in the other tubes. The last well was kept blank serving as control as no Curcumin was added to it. The reaction mixtures were then incubated in thermo cycler (Eppendorf) with following conditions: initial denaturation for 1 min at 95 °C, followed by 30 cycles of denaturation for 30 s at 95 °C, annealing for 30 s at 50 °C, extension for 1 minute at 72 °C and finally held at 4 °C following completion. The PCR products were then mixed with 6X orange loading dye and then resolved on 3% agarose gel pre-stained with ethidium bromide. The gels were at last visualized using analyzed on ImageQuant LAS 4000 (GE Healthcare).

Circular Dichroism spectroscopy assay

All the circular dichroism (CD) experiments were performed with the help of J-815 Spectropolarimeter (JASCO) instrument. The CD spectra experiments were carried out at the constant temperature of 25°C which was maintained using the peltier junction temperature controller. A constant supply of nitrogen was flushed into the cuvette containing chamber to avoid the condensation of water outside the cuvette. The spectrum for each experiment was measured using the quartz cuvette of 0.2cm path length containing 10.0 μM CGG and AU

RNA samples and titration were done with increasing concentrations of Curcumin in 50.0 mM KCl, 10.0 mM phosphate buffer (K+) at pH 7.0 and a scanning speed of 20nm/min between the wavelength of 200nm to 320nm. The RNA samples were heated at 95°C for 10mins and then slowly cooled down to the RT before each set of experiment in 10mM phosphate buffer (50mM KCl, pH 7.0). Buffer subtraction was done in each set of experiment to avoid signal contribution from the buffer. The data were plotted using the SigmaPlot 12.0 software both for the CD spectra and CD melting experiment.

RNA thermal denaturation study

RNA thermal denaturation experiments were carried out using J-815 Spectropolarimeter (JASCO) equipped with Peltier temperature programmer and water Peltier system PCB-1500. CGG and AU paired RNA (10.0 µM) were diluted in 1x potassium phosphate buffer (50.0mM KCl, 10.0mM phosphate buffer (K+) and heated at 90-92°C for 5-10 min then allow it to cool down at RT for next 30 min before start of the experiment. The Melting curve of RNAs were monitored from 25 °C to 95 °C at the rate of 1 °C/min with the titration of Curcumin till drug/nucleic acid ratio 2.0. The absorbance changes at 267 nm were normalized with similar buffer and plotted against temperature using the SigmaPlot 12.0 software.

Molecular Docking

The crystal structure of duplex CGG motif (PDB ID: 3JS2) were used for the docking.[1] The addition, replacements of residues and optimization of duplex RNA structure with Curcumin were built in Discovery studio 3.5 (Accelerys Inc.,USA) using CHARMm force field. Curcumin docking studies were performed on Autodock 4.0 where duplex RNA treated as solid rigid structure, RNA and Curcumin file prepared in PDBQT format. The other parameters kept as a default values during docking. CGG duplex RNA and Curcumin were converted to AD4 format files and Gesteiger charges were assigned to the atoms. The grid box was established in such way which covers the complete RNA structure so that Curcumin can explore the whole conformational space. The grid centre was placed by centring the grid box so that both minor groove or intercalation site utilise equally. Lamarckian genetic algorithm [2]was run to carry out energy evaluation of the location of the ligand according to target energy grid and the results were analysed based on binding energy. The best dock result was further processed to prepare image in Discovery studio 3.5[3].

Improvement of splicing defect by Curcumin in FXTAS cellular model

To determine whether Curcumin improve alternative splicing defect *in-vitro*, a FXTAS cellular model were used. Briefly, HEK293 cells were grown in 24 well plates as monolayer in the growth medium contain 1X DMEM, 10% fetal bovine serum, 1% antibiotic and antimycotic solution at 37 °C with 5% CO_2. After cell reached 80-90% confluency, HEK cell were transfected with the equal amount of plasmid contain CGGx99 repeat and targeted mini-genes (*SMN2* and *Bcl-x*) using Lipofectamine 3000 reagent (Thermo Fisher Scientific), according to standard manufacturers protocol. Transfection mixture was removed after 4-5 hours and Curcumin containing DMEM were added. After 24 hours, cells were lysed in the plate and total RNA were recovered using RNA isolation kit (Invitrogen), according to standard manufacturers protocol.

All different conditioned RNA sample were subjected to reverse transcribe (RT-PCR) using cDNA synthesis kit from Bio-Rad, followed standard protocol. Out of 500 ng reverse transcribed mRNA 100 ng were subjected for semi-quantitative PCR. The PCR protocol used: denaturation for 1 min at 95 °C, annealing for 1 min at 55 °C, extension for 2 min at 72 °C and final extension for 10 min at 72 °C. The PCR protocol was run for 25-30 cycles. The PCR product were analysed on agarose gel electrophoresis, stained with ethidium bromide and image were recorded using ImageQuant LAS 4000 (GE Healthcare). The splicing isoform intensity was quantified using imageJ software. The primer sequence mentioned in the table S4 for each construct[4].

Two control condition were used to study pre-mRNA splicing defect. 1) Targeted mini-genes (*SMN2* and *Bcl-x*) were co-transfected with plasmid that lack CGG repeat at 5′UTR as describe above. 2) Co-transfection of CGG repeat plasmid with mini-genes whose pre-mRNA splicing not govern by sam68 (cTNT)[4, 5].

Percentage cell viability enhancement of FXTAS cellular model with Curcumin treatment using MTT assay

To determine improvement of cell viability of FXTAS cell model in presence of Curcumin, MTT assay were performed. [3] The HEK cells (5 - 10 x 10^3 cells/well) were plated in the 96 well culture plates as mono layer in triplicate and allowed to grow in the complete media (1X DMEM, 10% FBS), at 37 °C with 5% CO_2. After cell were attached properly and reached to optimum confluence. Cell were transfected with pcDNA-EGFP plasmid (control), CGGx20

normal repeat plasmid and CGGx99 diseased plasmid using lipofectamine 3000 reagent from Thermo Fisher Scientific as per manufacturer protocol. Curcumin treatment was given after 4 hrs incubation of transfection for 24 hrs. After 24 hrs incubation, 10 µL of 5 mg/mL MTT in PBS was added in each well and further incubated for next four hours at 37 °C. The insoluble yellow coloured MTT into dark coloured formazon crystals by intracellular reduction. 100µL DMSO were added to dissolve the formazon crystal structure and microplate reader (Synergy™ H1 multi-mode microplate reader) were used for taking absorbance. Half-maximal inhibitory concentration (IC_{50}) of the Curcumin was determined by the using formula.

$$\% \ inhibition = \frac{Control\ absorbance - \ sample\ absorbance}{Control\ absorbance}\ x100$$

Quantification of RAN translation by western blot

HEK293 cells were seeded in 6 well plate in amonolayer form and transfected with 2.5µg of plasmid coding CGGx99-GFP using lipofectamine 3000 as per manufacterer protocol from Thermo Fisher Scientific. The transfection mixture was removed after 5 hrs incubation and added fresh Compound containing media and the cell were incubated for 24hrs at 37 °C. After incubation, cells were lysed using RIPA buffer 200 µL/well containing 1 µL of Halt Protease Inhibitor Cocktail from Sigma. Cellular proteins concentration were calculated using bradford assay. Same amount of protein were loaded in SDS-PAGE for saperation and transferred to a PVDF membrane. Blot were incuabted with antiFMR1-polyG antibody from merck millipore as a primary antibodies. After primary incubation anti-IgG-horseradish peroxidase conjugate used as the secondary antibody. A chemiluminscent signals was detected using Luminata Crescendo Western HRP substrate from merck millipore in ImageQuant LAS 4000 (GE Healthcare).

Quantification of canonical GFP and non canonical FMRpolyG-GFP protein Inclusions

In order to determine the effect of Curcumin on polyG-GFP inclusions two different construct were used that contain r(CGG)x_{99}-GFP and pcDNA-GFP. The above mentioned construct was designed and validated by Nicolas group earlier[4]. Breifly, HEK cell were seeded in six well plate in 10% DMEM media. After cell were reached 80-90 percent confluencuy Transfection were done with above mentioned plasmid using lipofectamine 3000

reagent from Thermo Fisher Scientific as per manufacturer standerd protocol. The transfection cocktail were removed after 4hrs incubation and fresh media were applied which contain respective Curcumin concentration and the cells were incbate for 18-24 hrs at 37°C. Cell after washing with PBS, fixed with 4% paraformaldehyde for 15 minutes. Image were captured and protein aggregates visualised at hihger magnification using flurosence confocal microscopy and processed to remove background signals. 100 positve transfected cell from each well were selected and manually graded as "with protein aggregates" and "no aggregates". The number of GFP protein inclusion were manually counted from three independent experiment and standerd deviation were calculated[6].

References

[1] A. Kumar, P. Fang, H. Park, M. Guo, K.W. Nettles, M.D. Disney, A Crystal Structure of a Model of the Repeating r(CGG) Transcript Found in Fragile × Syndrome, Chembiochem 12(14) (2011) 2140-2142.

[2] M.G. M., G.D. S., H.R. S., H. Ruth, H.W. E., B.R. K., O.A. J., Automated docking using a Lamarckian genetic algorithm and an empirical binding free energy function, Journal of Computational Chemistry 19(14) (1998) 1639-1662.

[3] A. Tawani, A. Amanullah, A. Mishra, A. Kumar, Evidences for Piperine inhibiting cancer by targeting human G-quadruplex DNA sequences, Scientific Reports 6 (2016) 39239.

[4] C. Sellier, F. Rau, Y. Liu, F. Tassone, R.K. Hukema, R. Gattoni, A. Schneider, S. Richard, R. Willemsen, D.J. Elliott, P.J. Hagerman, N. Charlet-Berguerand, Sam68 sequestration and partial loss of function are associated with splicing alterations in FXTAS patients, The EMBO journal 29(7) (2010) 1248-61.

[5] M.D. Disney, B. Liu, W.Y. Yang, C. Sellier, T. Tran, N. Charlet-Berguerand, J.L. Childs-Disney, A small molecule that targets r(CGG)(exp) and improves defects in fragile X-associated tremor ataxia syndrome, ACS Chem Biol 7(10) (2012) 1711-8.

[6] E. Khan, A. Tawani, S.K. Mishra, A.K. Verma, A. Upadhyay, M. Kumar, R. Sandhir, A. Mishra, A. Kumar, Myricetin Reduces Toxic Level of CAG Repeats RNA in Huntington's Disease (HD) and Spino Cerebellar Ataxia (SCAs), 13(1) (2018) 180-188.

Supplementary Tables

Table S1. The dissociation constant (K_d) values was determined from flouresecne titration assay of Curcumin with different **(5'CNG/3'CNG)x1** RNAs motif after two-mode curve fitting. K_d^1 denotes the first preferential binding and K_d^2 denotes the second preferential binding of Curcumin with RNAs.

**S. No.**	**RNA (5'CNG/3'GNC) x 1**	**K_d^1 (μM)**	**K_d^2 (μM)**
1.	(5'CAG/3'GGC) x 1	0.75 ± 0.08	3.59 ± 1.1
2.	(5'CCG/3'GAC) x 1	1.05 ± 0.07	1.58 ± 0.26
3.	(5'CGG/3'GAC) x 1	0.57 ± 0.11	4.2 ± 0.23
4.	(5'CCG/3'GUC) x 1	0.78 ± 0.10	5.17 ± 0.42
5.	(5'CAG/3'GCC) x 1	0.44 ± 0.04	4.77 ± 0.34
6.	(5'CUG/3'GCC) x 1	0.66 ± 0.04	1.91 ± 0.70
7.	(5'CGG/3'GCC) x 1	0.12 ± 0.02	0.59 ± 0.10
8.	(5'CAG/3'GAC) x 1	0.56 ± 0.07	1.45 ± 0.52
9.	(5'CCG/3'GGC) x 1	0.86 ± 0.10	1.5 ± 0.92
10.	(5'CUG/3'GUC) x 1	0.74 ± 0.01	24.1 ± 1.7
11.	(5'CAG/3'CUG) x 1	2.1 ± 0.12	3.94 ± 0.84

Table S2. The dissociation constant (K_d) values was obtained from flouresecne titration assay of Curcumin with different $r(CGG)^{exp}$ RNA, $r(CNG)^{exp}$ RNA, AU paired RNA and DNA controls after two-mode curve fitting. K_d^1 denotes the first preferential binding and K_d^2 denotes the second preferential binding of Curcumin with target RNAs and controls.

S. No.	DNA/RNA	K_d^1 (µM)	K_d^2 (µM)
**1.**	r(CGGx1)	0.12 ± 0.02	0.59 ± 0.23
**2.**	r(CGGx2)	0.13 ± 0.01	2.4 ± 0.98
**3.**	r(CGGx3)	0.12 ± 0.02	1.01 ± 0.40
**4.**	r(CGGx4)	0.106 ± 0.01	1.22 ± 0.29
**5.**	r(CGGx6)	0.092 ± 0.004	0.34 ± 0.07
**6.**	r(CGGx20)	0.034 ± 0.008	1.48 ± 0.45
**7.**	r(CGGx40)	0.029 ± 0.01	0.91 ± 0.08
**8.**	r(CGGx60)	0.013 ± 0.005	0.56 ± 0.11
**9.**	r(AUx6)	2.31 ± 0.36	14.1 ± 1.6
**10.**	r(CAGx6)	1.6 ± 0.180	6.3 ± 2.9
**11.**	r(CCGx6)	0.84 ± 0.05	14.3 ± 2.5
**12.**	r(CUGx6)	2.2 ± 0.3	9.2 ± 1.7
**13.**	Yeast t-RNA	3.5 ± 0.28	13.9 ± 4.4
**14.**	Ckit DNA	0.93 ± 0.17	19.1 ± 3.1
**15.**	Cmyc DNA	1.8 ± 0.36	27.9 ± 1.7
**16.**	Bcl2 DNA	1.2 ± 0.36	33.2 ± 2.4
**17.**	Tel22	1.6 ± 0.08	21.3 ± 4.3
**18.**	CT DNA	6.3 ± 0.48	54.6 ± 7.2

Table S3. Thermodynamic parameter values of different r(CGG)^{exp} RNAs, r(CNG)^{exp} RNAs & r(AU)$_6$ duplex RNA (control) and with Curcumin.

**Parameters**	**RNA**							
	**r(CGGx6)**	**r(CGGx20)**	**r(CGGx40)**	**r(CGGx60)**	**r(AUx6)**	**r(CAGx6)**	**r(CCGx6)**	**r(CUG6)**
N1 (sites)	0.36 ± 0.06	0.34 ± 0.03	0.309 ± 0.08	0.265 ± 0.07	0.369 ± 0.11	0.582 ± 0.14	0.75 ± 0.72	0.77 ± 0.20
K1 (M^{-1})	1.7E6 ± 1.2	3.8E7 ± 1.2	8.5E7 ± 1.4	8.6E7 ± 0.97	7.1E4 ± 0.81	6.8E4 ± 1.8	4.6E4 ± 0.88	3.2 E4 ± 0.46
ΔH1 cal/mol	-2.1E5 ± 0.48	-4.2E5 ± 0.01	-4.1E5 ± 3.2	-2.7E5 ± 0.73	-4.7E6 ± 4.53E5	-1.7E4 ± 0.37	-3.4E4 ± 1.9	4.6E5 ± 3.8
ΔS1 cal/mol/deg	-693.6 ±160	-1.3E3 ± 0.04	-393.2 ± 216.5	-906 ± 245.2	-2.6E4 ± 1.3E3	-52.1 ± 16.7	-394.5 ± 359.9	5.76E3 ± 0.77
N2 (sites)	178.9 ± 160	0.28 ± 0.11	11.1 ± 6.0	6.8 ± 2.5	0.44±0.05	1.03 ± 0.96	171.5 ± 30.4	0.53 ± 0.16
K2 (M^{-1})	1.3E4 ±7807	1.9E5 ± 0.31	3.2E5 ± 1.5	3.5E5 ±3.8	2.5E4 ±1.0	2.5 ± 0.89	5.2E3 ± 2.2	8.1E4 ± 2.6
ΔH2 (cal/mol)	-7852E7 ± 575	-3.0E6 ± 2.8	-2.4E4 ± 0.53	-3.9E6 ± 4.1	-1.4E5 ± 0.37	-0.52E5 ± 1.5	-3.3E4 ± 1.7	-83.7 ± 39
ΔS2 (cal/mol/de)	-7.6E4 ± 2.2	-3.8E3 ± 1.8	-45.2 ± 35.1	-15.5E5 ± 21	319±251	1.77E5 ± 0.53	-105.85 ± 75	-673.5 ± 27.5

Table S4. PCR primer sequences for splicing defect.

S no.	Gene	Forwards primer	Reverse primer
**1.**	SMN2 mini-gene	5’GGTGTCCACTCCCAGTTCA A	5’ GCCTCACCACCGTGCTGG
**2.**	Bc l-x mini-gene	5’GGAGCTGGTGGTTGACTTT CT	5’ TAGAAGGCACAGTCGAGG
**3.**	cTNT mini-gene	5’GTTCACAACCATCTAAAGC AAGATG	5’ GTTGCATGGCTGGTGCAGG
**4.**	β-Actin	5’ CCTGGCACCCAGCACAAT	5’ GGGCCGGACTCGTCATAC
**5.**	(CGG)99-GFP	5’GCACGACTTCTTCAAGTCC GCCATGCC	5’GCGGATCTTGAAGTTCACCTT GATGCC

Supplementary Figures

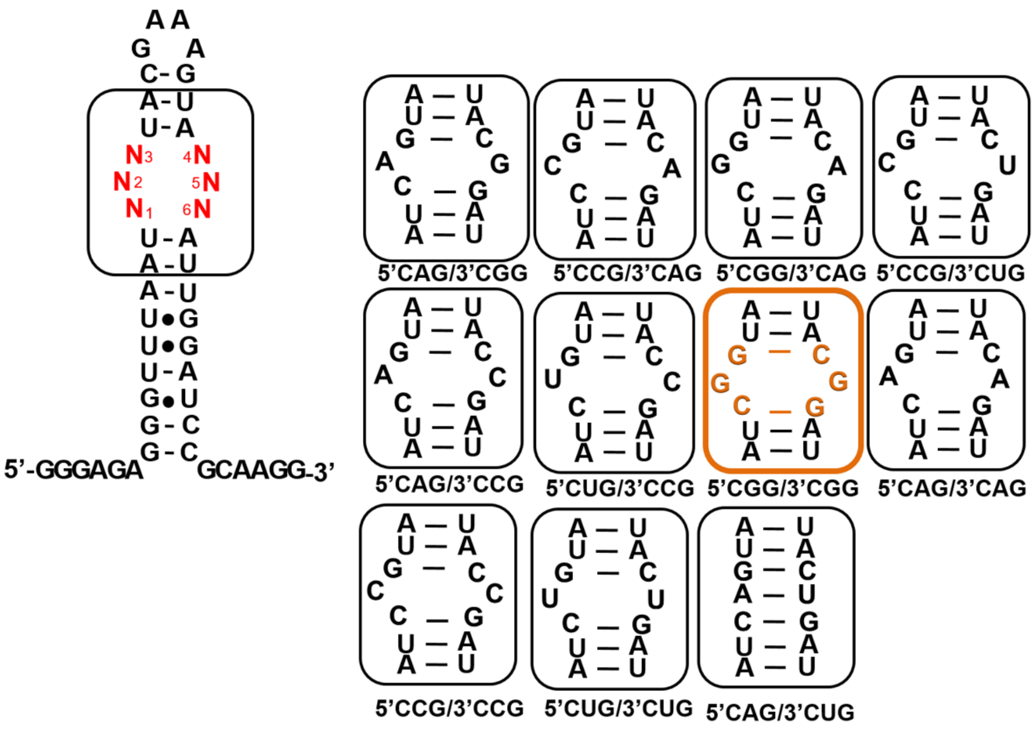

Figure S1. Secondary strucutre of different 1x1 internal nucleotide motif (**5'CNG/3'GNC**) of RNAs sequence.

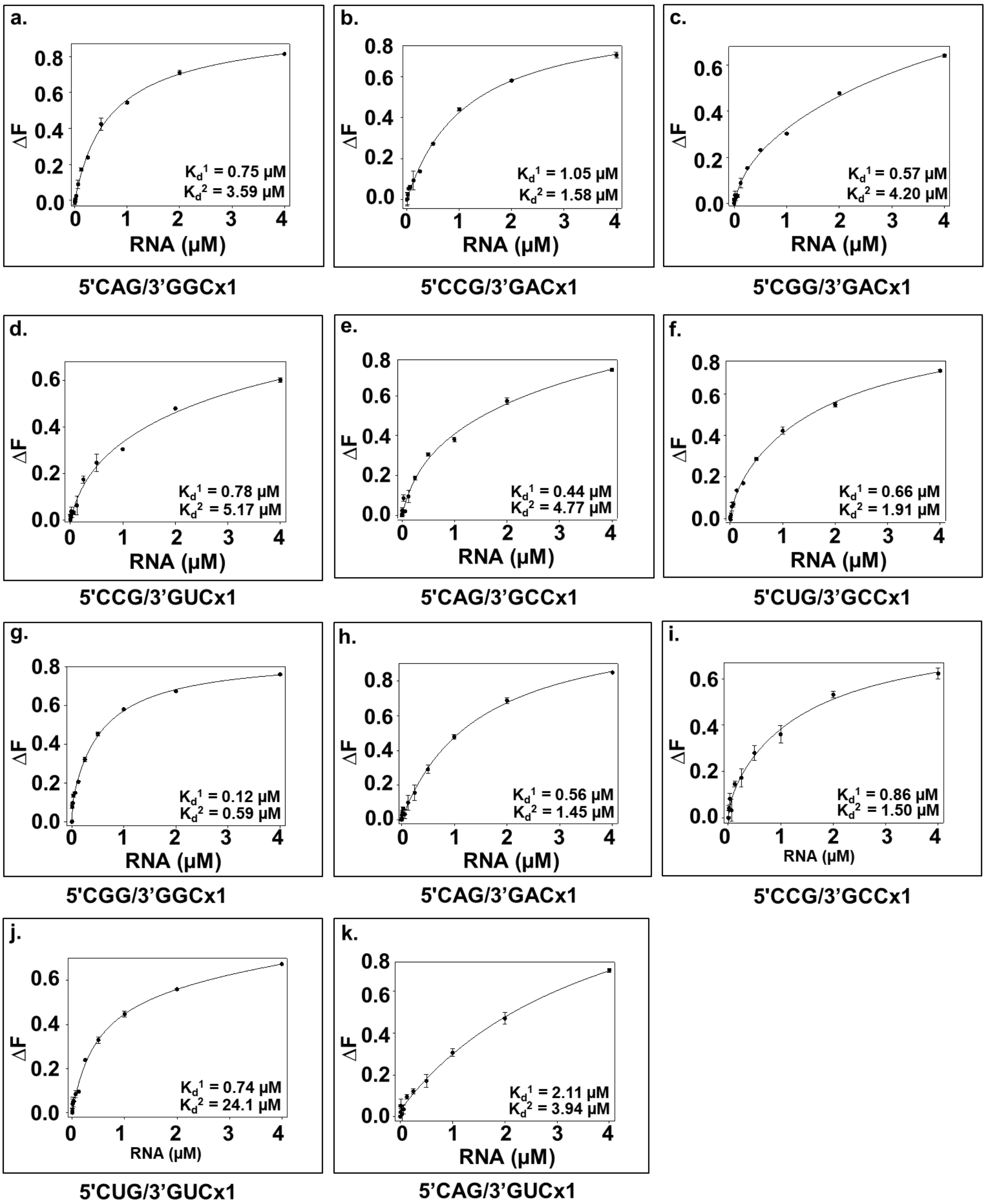

Figure S2. Schematic representation of fluorescence titration assay plots of different 1 x 1 **(5′CNG/3′GNC)** RNAs motif with Curcumin. Solid line represents the two-mode fitting. The dissociation constant (K_d^1 and K_d^2) are mentioned below.

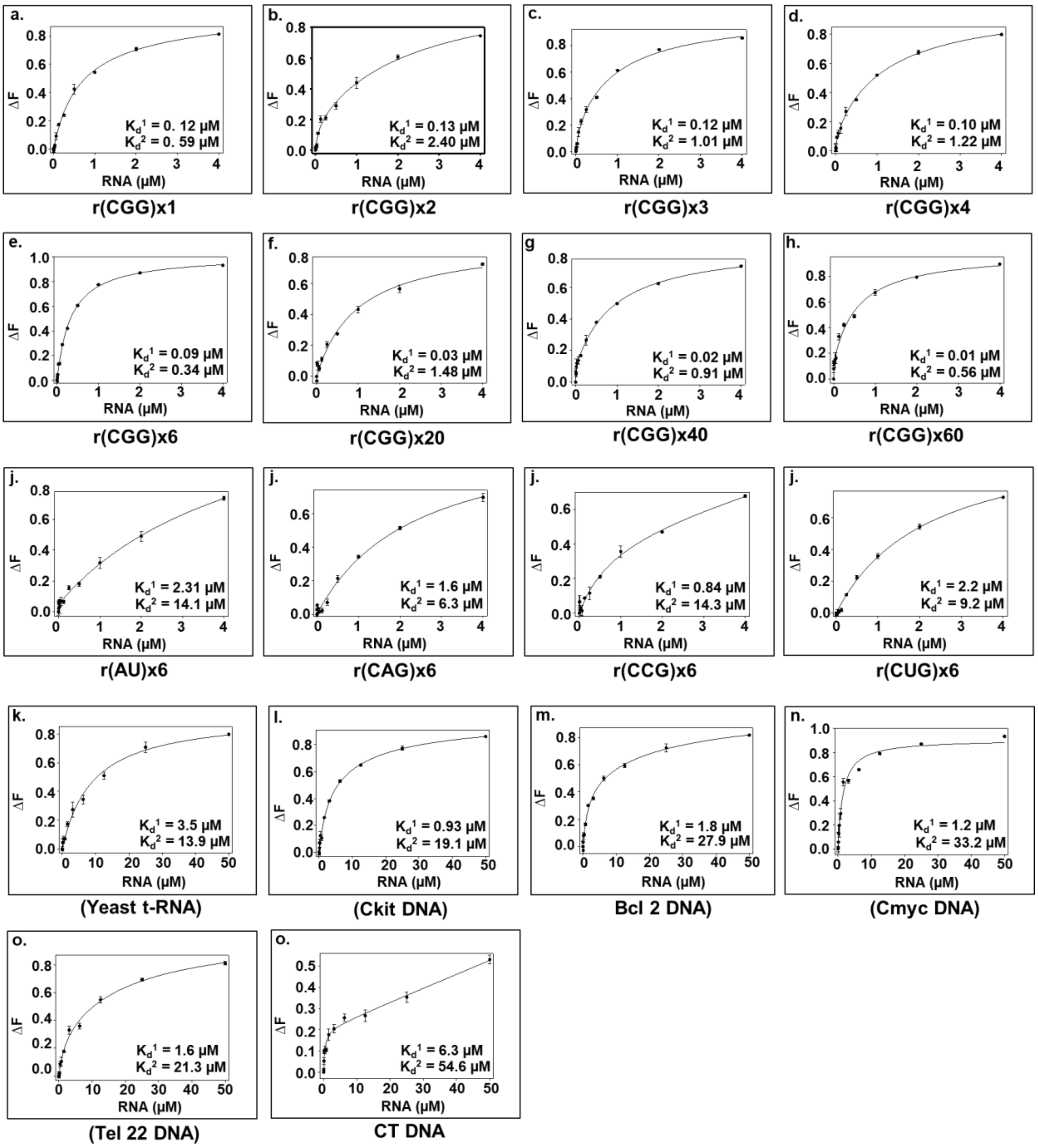

Figure S3. Diagram shows the fluorescence titration assay plots of Curcumin with different CGG repeat RNAs and control RNAs (AU)6x, r(CAG)6x, r(CCG)6x & r(CUG)6x) & DNA controls (c-kit DNA, cmyc DNA, bcl2 DNA, tel22 DNA & ct DNA). Curve represents the two-mode fitting. The K_d^1 and K_d^2 are mentioned below.

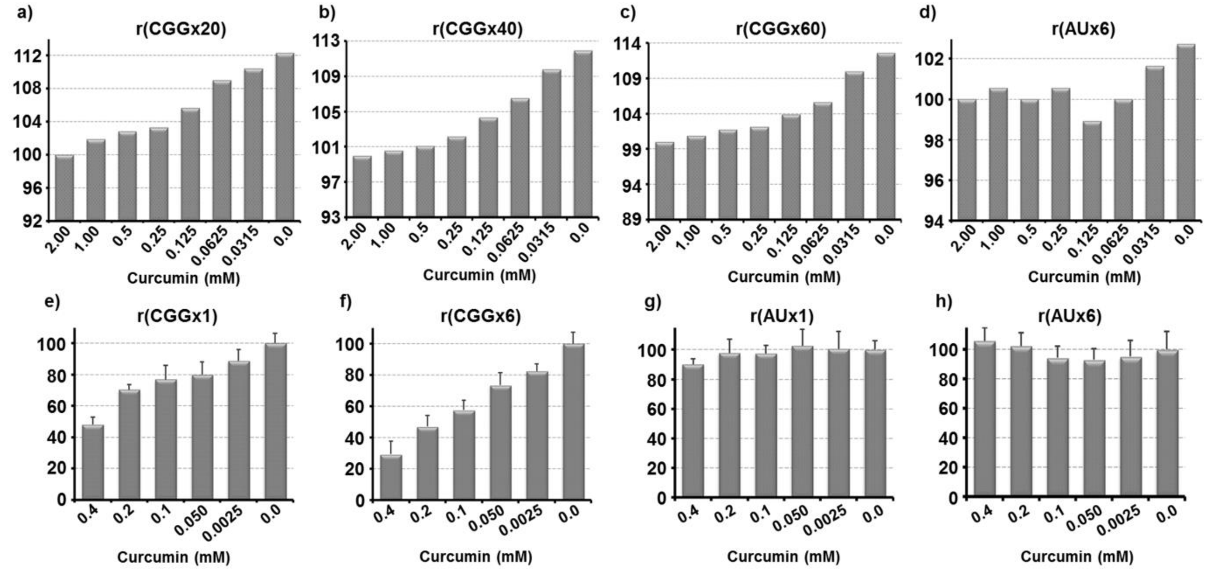

Figure S4. **(a-d)** Bargraph represent the precentge distance migration calculation of CGG repeats RNAs and AUx6 RNA in the presence of Curcumin. **(e-h)** Plot represents quantification of CGG DNA template PCR amplification inhibition with varied Curcumin concentration.

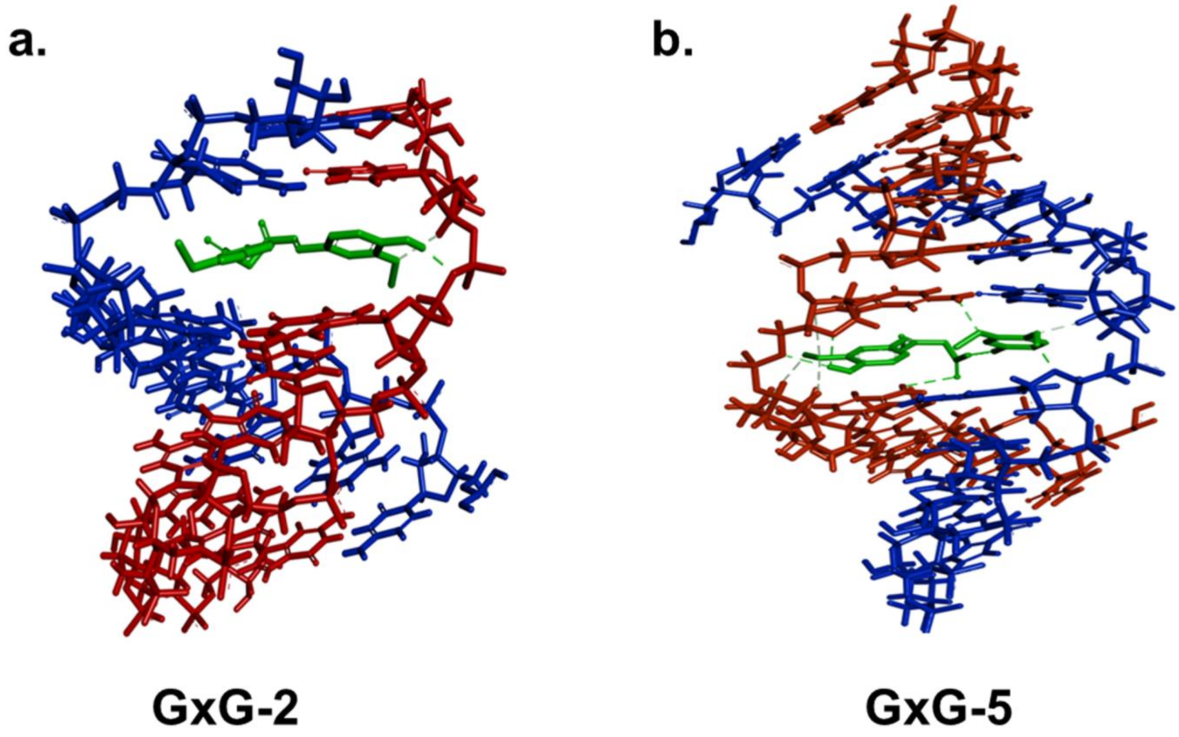

Figure S5. Curcumin docking study with CGGx3 RNA (PDB ID: 3JS2). **a)** Represent the dock image of GxG-2 position. **b)** Represent the dock image GxG-5 position. The best binding energy of GxG-2 & GxG-5 is -7.35 and -6.71 kcal/mol respectively

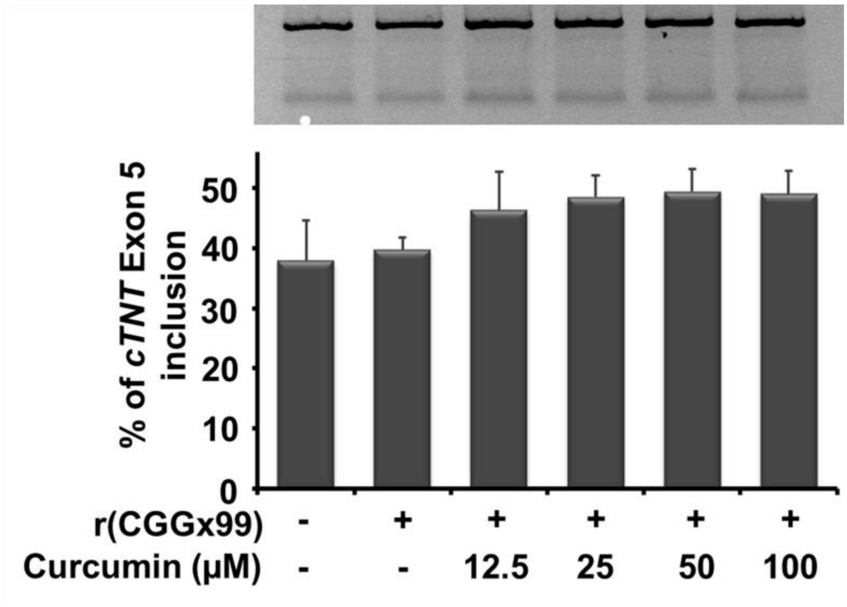

Figure S6. Gel image (top) and quantification of alternative pre-mRNA splicing defect of cTNT mini-gene in CGGx99 transfected and healthy cells as a function of Curcumin concentration. Briefly, cTNT and CGGx99 plasmid (toxic) co-transfected in HEK293 cells. curcumin does not affect alternaive splicing defect of cTNT mini-gene.

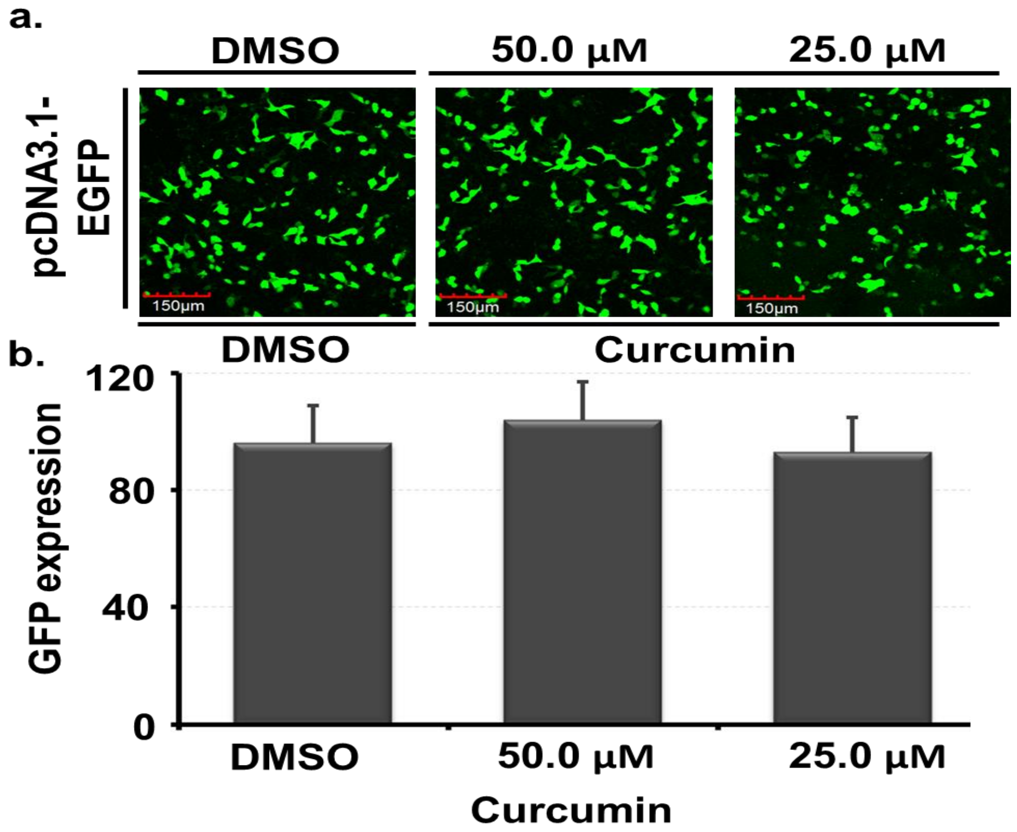

Figure S7. (a) Diagram showing the EGFP expression of pcDNA3.1 plasmid (lacking r(CGG)^{exp}) of HEK293 transfected cell in absence and presence of Curcumin. **(b)** Bar graph represents the normalized EGFP intensity of Curcumin treated and untreated cells.

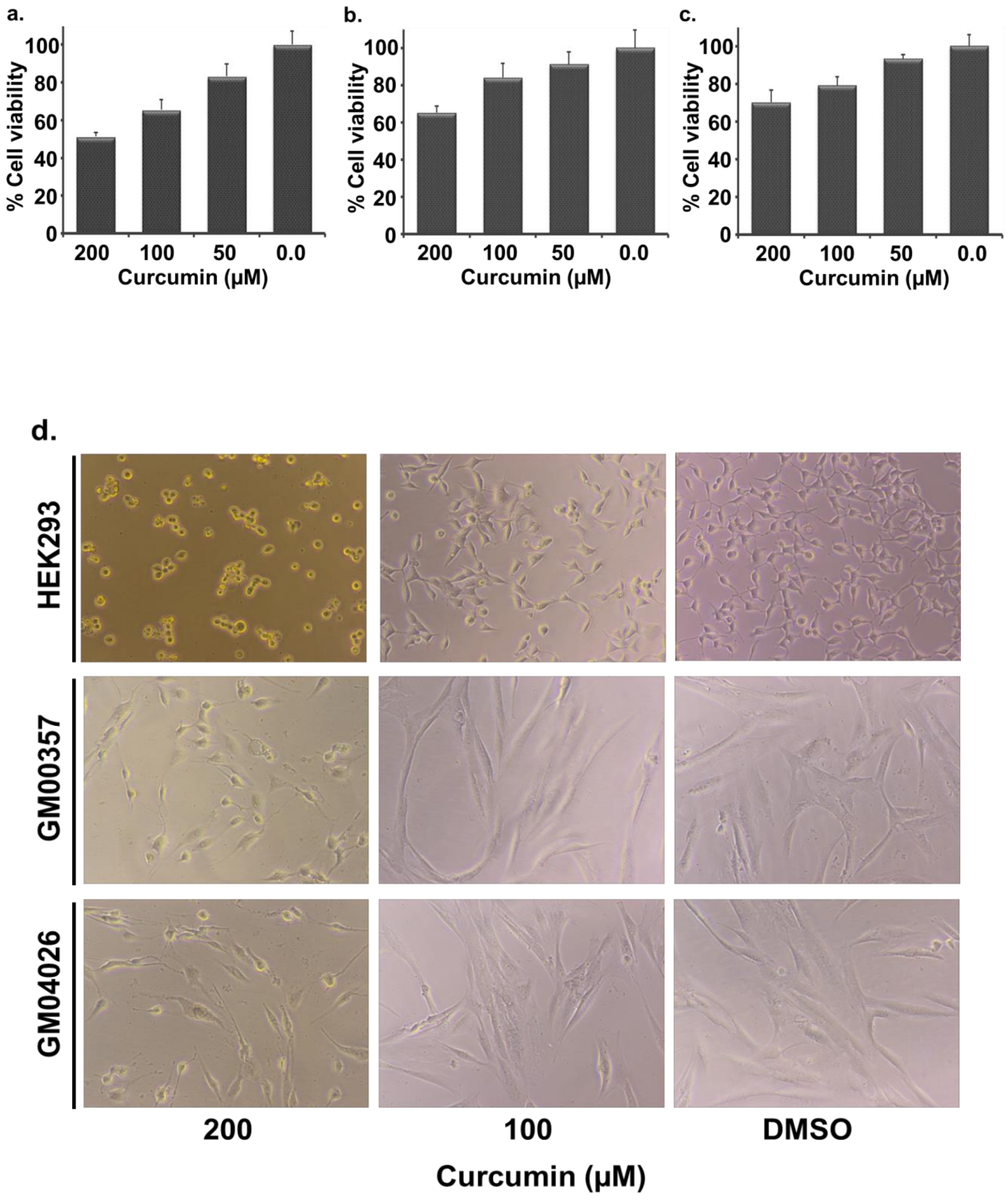

Figure S8. Plot showing percentage cell viability of different established cell lines including **(a)** HEK293, **(b)** GM00357, and **(c)** GM04026. **d)**. Image showing cellular morphology in treated and untreated condition.

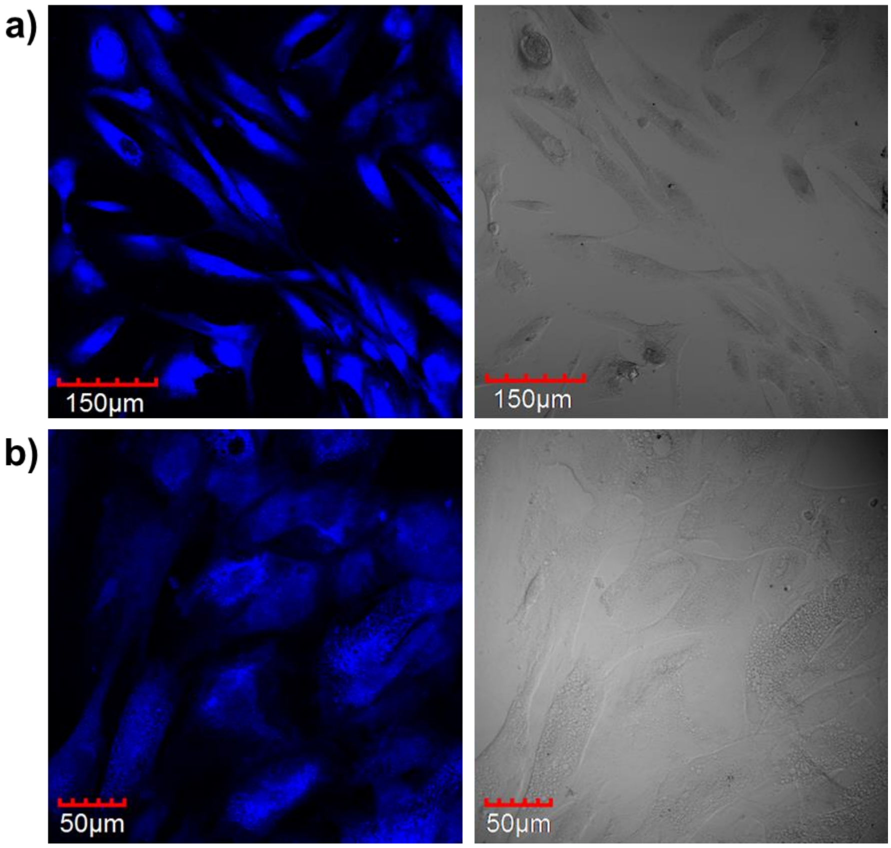

Figure S9. Cellular intake of Curcumin in patient derived cells (GM04026). **a)** 20x magnification **b)** 40x magnification.
